# The Effect of N‑Donor Ligands on Activation of [Ru(tpy)(L–py)(AcCN)]^2+^ Precatalysts in CO_2_ Reduction Reaction Electrocatalysis

**DOI:** 10.1021/acs.inorgchem.6c02082

**Published:** 2026-06-18

**Authors:** Maurício Portiolli Franco, Giovana Vitiello Teixeira, André Luiz Barboza Formiga

**Affiliations:** 344102 Instituto de Química − UNICAMP, Caixa Postal: 6154, CEP: 13083-970 Campinas, SP, Brazil

## Abstract

The electrocatalytic reduction of CO_2_ to CO in anhydrous media requires the formation of the M–CO_2_ bond. Using [Ru­(tpy)­(bpy)­S]^2+^ as a model, and substituting a pyridine of bipyridine­(**bpy**) for L = 123-triazole­(**123-trz**), oxazole­(**oz**), pyrrole­(**pyrr**), pyrazole­(**pr** **z**), or NHC was computationally studied using TPPSh/def2-svp for geometries, SP correction with def2-tzvp, and implicit acetonitrile solvation. Complexes with **pyrr**, **prz**, and **NHC** undergo both reductions on the tpy ligand, shifting the second reduction to more negative potentials compared to **bpy**, **123-trz**, and **oz** (which are at least 0.15 V more positive). For the latter, the second reduction occurs on the bidentate ligand generating [Ru­(tpy^·^)­(py-L^·^)­AcCN]^0^. The general reaction path features two reductions, solvent dissociation to a pentacoordinated complex, and CO_2_ association, except for **NHC,** where theoretical and experimental data suggest dissociation after the first reduction. Dissociation barriers varied from 19.0 (**oz**) to 12.9 kcal/mol (**prz**), while association barriers ranged from 9.0 (**NHC**) to 3.8 kcal/mol (**oz**). Finally, the *cis*/*trans* product ratio from CO_2_ association is directly influenced by the τ_5_ distortion parameter, serving as a qualitative indicator of selectivity.

## Graphical abstract

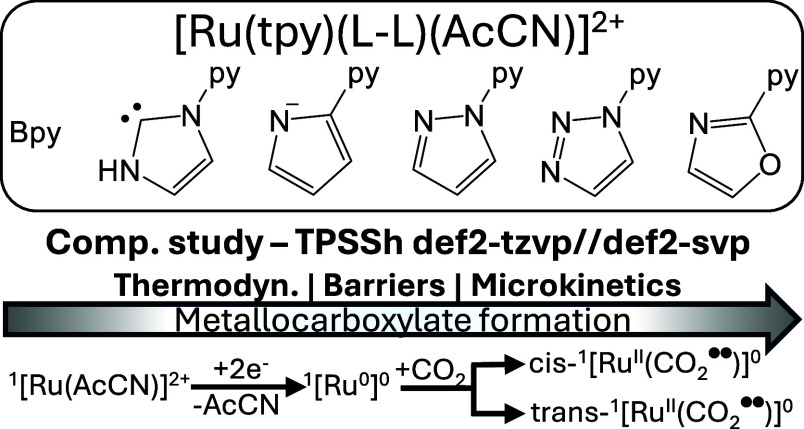

## Introduction

Carbon dioxide emissions arise from the most diverse human activities and present a serious environmental issue given it is the most common greenhouse gas with emissions of over 37 billion tons in 2021.^1^ Aiming to lessen the impact of CO_2_ emissions, one solution is to transform this low industrial value feedstock into products of greater added value, such as carbon monoxide,^2^ formate,^3^ carbonate,^4^ and other C_2_ products, for example, ethanol,^5,6^ through its reduction.^7,8^ The problem of the CO_2_ reduction reaction (CO_2_RR) to produce valuable chemicals is the high stability of the linear triatomic molecule, which requires a potential of −2.14 V versus SCE, pH 7.0, for a one-electron reduction.^9,10^ However, ruthenium polypyridine complexes, such as [Ru­(tpy)­(bpy)­(AcCN)]^2+^, can act as electrocatalysts, facilitating the electron transfer to CO_2_.^11^

The first report of CO_2_RR with [Ru­(tpy)­(bpy)­(AcCN)]^2+^ was presented by Nagao et al. in 1994 ^12^, and later, its kinetics were studied by Meyer and collaborators,^13^ suggesting a rate constant of 2–3 s^–1^ with CO as the major product. Substitution of one pyridine ring in **bpy** for 1-methyl-benzimidazolium (**Mebim**) to enhance a stronger *t* *rans*-effect increased the rate constant to 19 s^–1^. The proposed initial step to CO_2_RR is an EEC mechanism (E = electron transfer step and C = chemical step), which means that two reductions take place before a chemical reaction occurs (Figure 1). The first reduction is centered on the terpyridine ligand and the second reduction depends on the bidentate ligand, as described experimentally for **bpy** and **Mebim**.^13^ In the former ligand, the second electron resides in the bidentate ligand, and the latter ones resides in the terpyridine once again.

**Figure 1:**
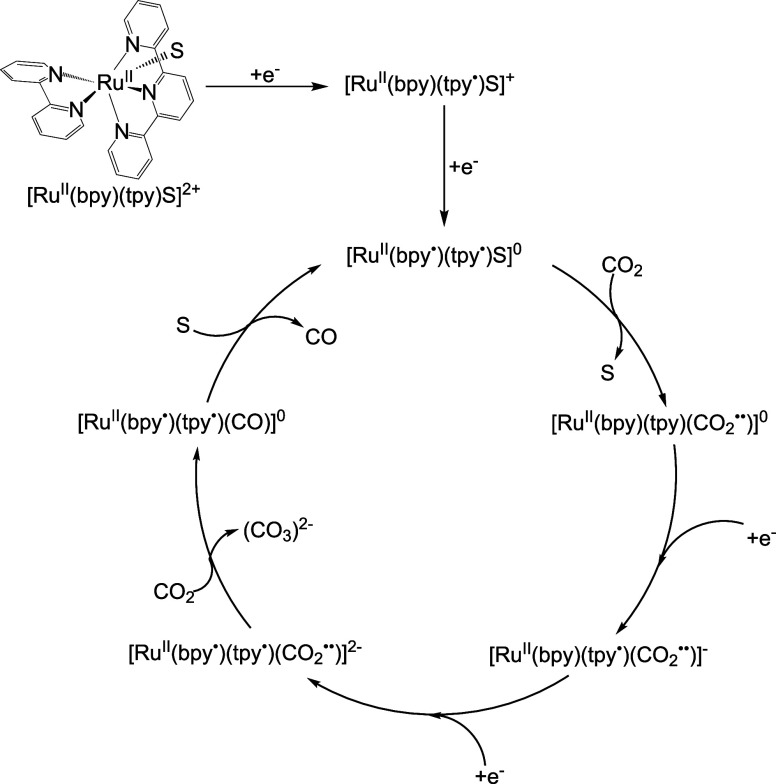
Proposed catalytic cycle to CO_2_ reduction reaction in an anhydrous medium. Adapted with permission from ref 14.

One crucial intermediate in CO_2_RR to produce CO is the metallocarboxylate species, which contains the M–C bond and undergoes dehydration in the presence of water or abstraction of O^2–^ to reach the carbonyl complex. The metallocarboxylate intermediate is formed by the dissociation of acetonitrile (eq 1) in the doubly reduced complex followed by CO_2_ association (eq 2). Queyriaux et al.^15^ and Meyer and co-workers^13^ separately reported that solvent dissociation is the rate-determining step for CO_2_RR to CO by [Ru­(tpy)­(bpy)­(AcCN)]^2+^ in an anhydrous medium.

$$[\mathrm{Ru}(\mathrm{tpy})(\mathrm{py}‐L)(\mathrm{AcCN}){]}^{0}\overset{k_{D}}{\underset{k_{-D}}{⇌}}[\mathrm{Ru}(\mathrm{tpy})(\mathrm{py}‐L){]}^{0}+\mathrm{AcCN}$$ 1

$$[\mathrm{Ru}(\mathrm{tpy})(\mathrm{py}‐L){]}^{0}+{\mathrm{CO}}_{2}\overset{k_{A}}{\to }[\mathrm{Ru}(\mathrm{tpy})(\mathrm{py}‐L)({\mathrm{CO}}_{2}^{\cdot \cdot }){]}^{0}$$ 2

The effect of the substituent on **bpy** can change the mechanism to ECE^8^ as observed when utilizing 2-methyl-bipyridine and the ^t^Bu-tpy ligand.^16^ This study utilizes density functional theory (DFT) calculations to comprehensively evaluate five bidentate ligands, each comprising a pyridine ring and a five-membered heterocycle. We will determine their reduction potentials and corresponding assignments, as well as investigate the stability and formation of their possible geometric isomers (*cis* and *trans*). These investigations will provide the necessary thermochemistry and kinetic theoretical data required for the formation of the critical target complex [Ru^*II*^(tpy)­(py-L)­(CO_2_^2–^)]^0^.

To understand the effect of bidentate ligands with properties distinct from those of the **bpy** complex such as negatively charged donor atoms and the effect of the position or type of heteroatoms in dissociation activation, the rate-determining step, five 5-membered heterocyclic rings bonded to pyridine ligands were evaluated toward the formation of metallocarboxylate and are presented in Figure 2. Bipyridine^13,16,17^ and N-heterocyclic carbene^18,19^ are well-described complexes in the literature. To the best of our knowledge, the other four complexes investigated in this work have not been previously reported. The oxazoline ligand has only been reported in its benzo analogue.^20^ Pyrrole ligands have been described with substituents on the five-membered ring.^21^ Pyrazole ligands have been reported mainly as pincer systems with ring substituents.^22,23^ Finally, 1,2,3-triazole ligands have been reported only in the [Ru­(bpy)_2_] motif.^24,25^

**Figure 2:**
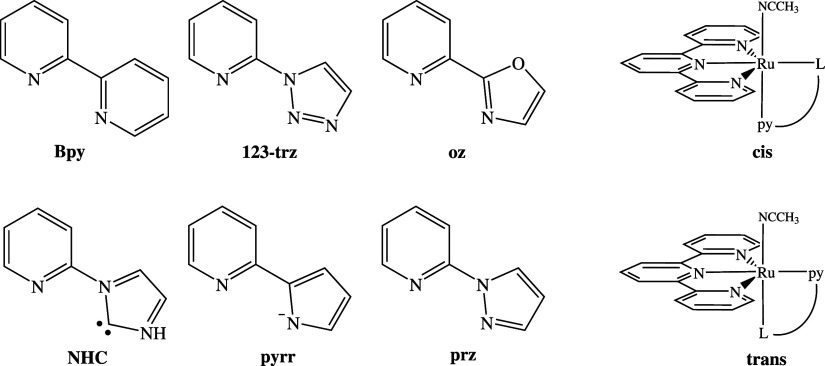
2D structures of bidentate ligands and their generic complexes, *trans*- and *cis*-[Ru­(tpy)­(py-L)­S]^2+^.

## Computational Details

Optimizations were performed using the ORCA 5.0.3 ^26^ quantum package employing the functional TPPSh^27^ and the basis set def2-svp,^28^ and single-point energy correction calculations were performed using the basis set def2-tzvp,^28^ its associated pseudopotential for the ruthenium atom,^29^ and the set of auxiliary bases def2/J.^30^ The TPPSh functional was selected because it has been shown to reproduce the correct spin-state ordering in spin-crossover systems.^31^ In addition, it has been reported to provide reliable molecular geometries when used with the chosen basis set.^32^ Dispersion effects were included through the D3BJ^33,34^ scheme, and implicit acetonitrile solvation was included by the CPCM model.^35^ The optimizations of intermediates and transition states were confirmed by Hessian calculations with no imaginary frequencies or only one imaginary frequency, respectively. The intrinsic reaction coordinates for all transition states were calculated to connect them to their reactants and products.

The correction from the 1 atm standard (G*) Gibbs free energy to the 1 mol/L standard (*G* ^o^) Gibbs free energy was made by adding 1.89 kcal/mol to the G* of each molecular species.^36^

$$G_{\mathrm{solv}}^{o}=G_{\mathrm{solv}}^{*}+\Delta G^{1\mathrm{atm}\to 1\mathrm{mol}/L}$$ 3

The dissociation (eq 1) and association (eq 2) reactions were simulated by microkinetics considering that reduction events do not have an activation barrier; i.e., the reaction equation starts from the doubly reduced complex.

The rate constants were calculated using the Eyring equation (eq 4), with Δ*G*_solv_^‡^ obtained by DFT calculations and used as input in microkinetic simulation of the reactions from eqs 1 and 2 in COPASI 4.44.^37^ The initial concentrations of [CO_2_] = 0.28 mol/L, [Ru­(tpy)­(py-L)­S]^0^ = 1.0 mmol/L, and the solvent were treated as being in its standard state, i.e., [CH_3_CN] = 19.15 mol/L. All of these concentrations were selected to reproduce the CO_2_RR experiment reported by Chen et al.^13^

$$k=\frac{k_{B}T}{h}\exp\left(\frac{-\Delta G_{\mathrm{solv}}^{‡}}{RT}\right)$$ 4

For a reduction reaction, Δ*G*_solv_^*^ is obtained fromeq [Disp-formula eq5]

$$A_{\mathrm{solv}}^{x}+ne^{-}\overset{\Delta G_{\mathrm{solv}}^{*}}{\to }A_{\mathrm{solv}}^{x-n}$$ 5

where *A*_solv_^*x*^ and *A*_solv_^*x*–*n*^ are the oxidized and reduced species, respectively, *ne* ^–^ is the number of electrons in the reduction process, and Δ*G*_solv_^*^ is calculated as

$$\Delta G_{\mathrm{solv}}^{*}=\Delta G_{\mathrm{solv}}^{*}(A^{x-n})-\Delta G_{\mathrm{solv}}^{*}(A^{x})-G_{\mathrm{gas}}^{o}(e^{-})$$ 6

where Δ*G*_solv_^*^ is the Gibbs free energy of the reduction reaction under standard conditions and *G*_gas_^o^(*e* ^–^) is the gas Gibbs free energy of an electron at 298 K (*G*_gas_^o^(*e* ^–^) = 0.867 kcal/mol).^38^ Then, standard reduction potentials (*E^o^*) were calculated using the Nernst equation (eq 7).

$$E^{o}(V)=-\frac{\Delta G_{\mathrm{solv}}^{*}}{nF}-\mathrm{SHE}$$ 7

Here, *n* is the number of electrons in the reduction process, *F* is the Faraday constant as 1 eV/V, and SHE is the absolute potential of the standard hydrogen electrode value (4.281 V).

## Results and Discussion

Our calculations are in agreement with reduction potentials and reduction assignments reported in the literature using cyclic voltammetry (CV) experiments for the standard complex [Ru­(tpy)­(bpy)­S]^2+^. Theoretical values for ^2^E_1_ and ^3^E_2_, where the superscript denotes the spin multiplicity of the electronic state and the subscript denotes the first or second reduction, were −1.03 and −1.47 V compared to the experimental values of −1.00 and −1.30 V. In addition, the assignments obtained from DFT and CV were identical for the first and second reductions and were assigned to terpyridine and bipyridine ligands, respectively.^13^

For the bpy complex, the dissociation occurs in the second reduction with a Δ*G*_D_^‡^ = 17.2 kcal/mol and Δ_R_ *G*_D_ = 8.0 kcal/mol. These values were obtained considering the product as a closed-shell singlet rather than a triplet state, with a triplet–singlet gap (Δ_T–S_) higher than zero, where positive values indicate singlet states and negative values indicate triplet states; the [Ru­(tpy)­(bpy)]^0^ bipyramidal complex has Δ_T–S_ of 0.27 eV. For this reason, dissociation energies (Δ_R_ *G*_D_) were calculated considering the forbidden spin-transition.^39^ The forbidden spin-transition type of reaction was also described in water oxidation catalysis with ruthenium complexes.^40^

A relevant change in the electronic structure of the dissociation product ^1^[Ru­(tpy)­(bpy)]^0^ compared to its predecessor is the reduction of Ru^*II*^ →Ru^0^. This is expected for this geometry, as exemplified by the examples [Fe^0^(CO)_5_],^41^ [Ru^0^(PPh_3_)_3_(CO)_2_],^42^ and [Os^0^(CO)_4_(η^2^-C_2_H_4_)].^43^ This charge transfer from the reduced **bpy** and **tpy** ligands to a molecular orbital with a Ru–N­(tpy) π character (Figure S1a) shows the reduction of the metallic center to zero. Considering the closed-shell state and geometry of the product, the ruthenium(0) species is the cause for the fast association step and the attack on the carbon atom in CO_2_. The orbitals involved in the electronic transfer from the doubly reduced solvatocomplex to the metallocarboxylate are presented in Figure S1a–f for the **bpy** ligand system, as well as for the other ligands studied in this work.

The interchange mechanism was also evaluated. The structure of the interchange TS occurs in the triplet state and has both monodentate ligands distant from the metallic center, with Ru–O from CO_2_ at 3.00 Å and Ru–N from acetonitrile at 4.49 Å, indicating a dissociative interchange mechanism (Id) (Figure 3). The simultaneous dissociation of AcCN and the approximation of CO_2_ occurs with a barrier height of 22.4 kcal/mol, producing a complex with Ru–OCO in the triplet state instead of the metallocarboxylate. Although the activation barrier is not prohibitive, the distance between the metallic center and the solvent indicates that dissociation should occur before CO_2_ association. Considering that the interchange mechanism has a barrier 5.2 kcal·mol^–1^ higher than that of the dissociative mechanism, this difference would translate into $\frac{k_{D}}{k_{\mathrm{Id}}}=6.81\times 10^{3}$ . Furthermore, the Ru–AcCN distance of 4.49 Å is sufficiently long to neglect any long-range interaction, indicating that this interchange transition state is not truly concerted. For these reasons, we considered only the dissociative pathway throughout this work.

**Figure 3:**
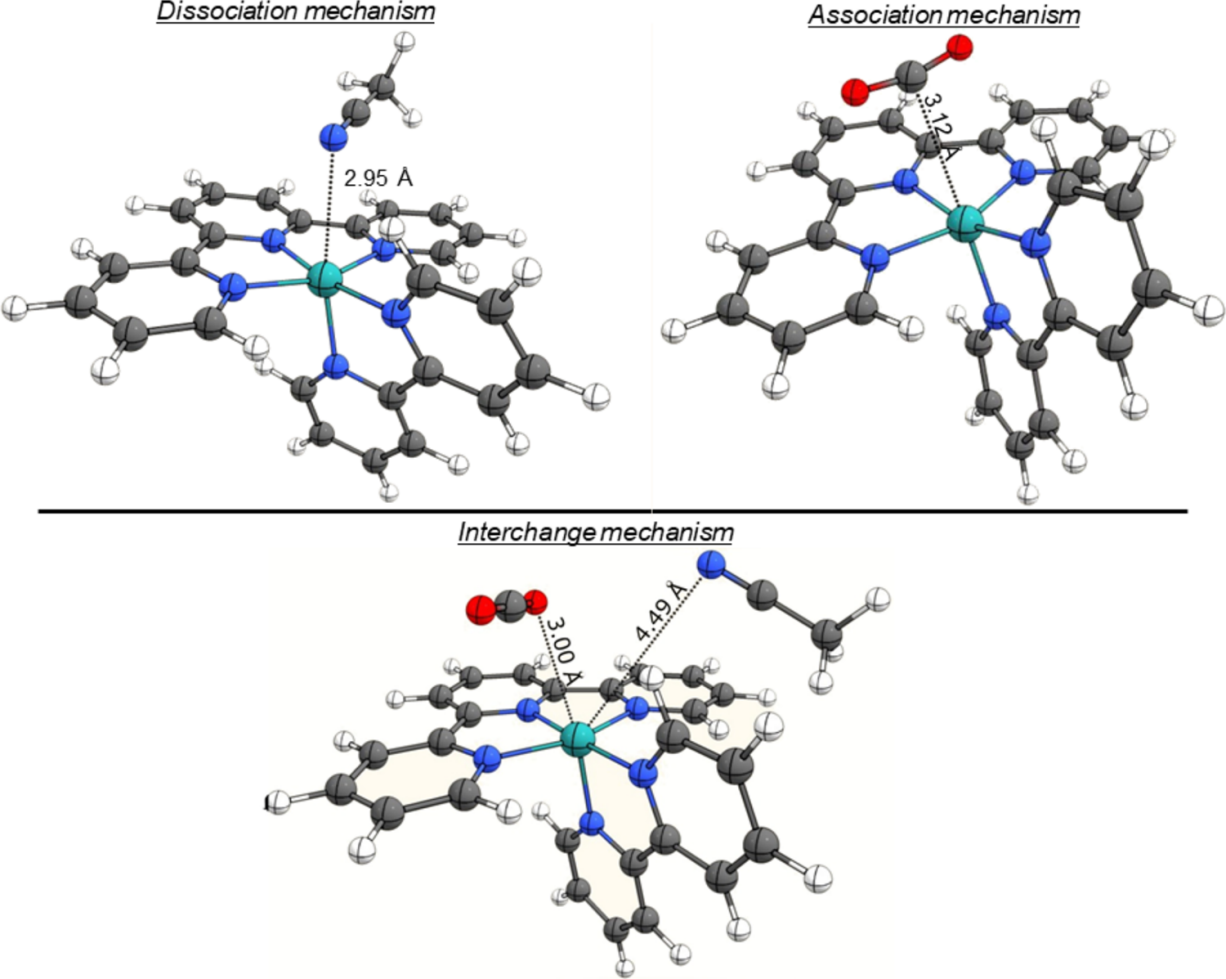
Transition state structures for the dissociative path (top) and for the interchange mechanism (bottom).

Microkinetics simulation of [Ru­(tpy)­(bpy)­(AcCN)]^0^ was carried out to evaluate the time evolution of concentrations of the species involved in metallocarboxylate formation (Figure 4). Since the equilibrium of acetonitrile dissociation is strongly shifted toward the reactants (*k*_D_ < *k*_–D_) and the association of CO_2_ is faster than *k*_–D_, the concentration of [Ru­(tpy)­(bpy)]^0^ is extremely low, with the highest concentration equal to 3.9 × 10^–12^ mmol/L, immediately after the start of the simulation because it did not start from chemical equilibrium. These results are in agreement with the conclusion of Meyer and collaborators,^13^ although their conclusion was for the full CO_2_RR and not just for the formation of the metallocarboxylate.

**Figure 4:**
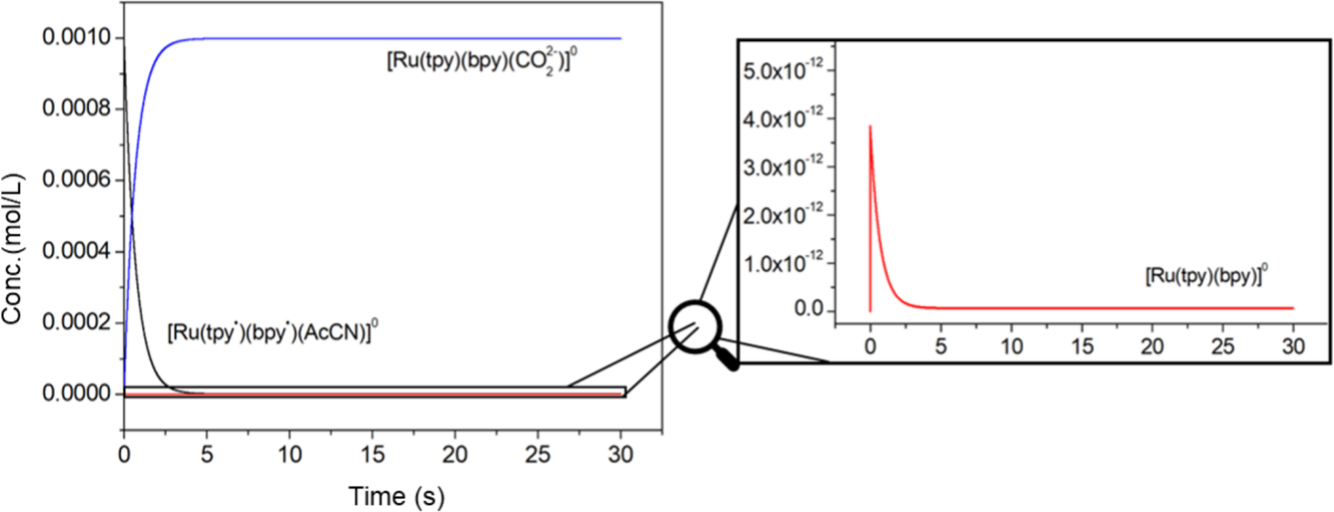
Kinetic simulation of precatalysis for the complex [Ru­(tpy)­(bpy)­(AcCN)]^0^.

Since the symmetry of bipyridine is broken due to the substitution of one of its rings, two position isomers are expected, one with the five-membered ring *trans* to acetonitrile and another with the ring in the *cis* position. In the particular case of the **NHC** ligand, we used a model with N–H instead of N–R to ensure the same steric bulk toward all ligands; yet, we also calculated the real N–Me system, and the results are provided in the Supporting Information.

All complexes have the first reduction located on terpyridine, and almost all potentials occur near −1.05 V versus SHE, except for the only negatively charged ligand, pyrrole, with ^2^E_1_ = −1.29 V (Table 1), as expected for the electrochemical ligand series reported by Lever.^44^ For the **NHC** ^18^ and **oz** ^25^ that have a benzo analogue as reported in the literature, the ^2^E_1_ values are in good agreement with both potential values.

**Table 1:** Reduction Potentials in Volts (V) for *Cis* and *Trans* Isomers of [Ru­(tpy)­(py-L)­S]^2+^ Calculated with TPSSh/def2-tzvp//TPSSh/def2-svp, D3BJ Dispersion Corrections, and Acetonitrile as the Implicit Solvent

		NHC		pyrr		prz		123-trz		oz	
ligands isomers	bpy	*cis*	*trans*	*cis*	*trans*	*cis*	*trans*	*cis*	*trans*	*cis*	*trans*
^2^E_1_	–1.03	–1.07	–1.11	–1.29	–1.29	–1.04	–1.06	–0.98	–1.02	–1.02	–1.04
^3^E_2_	–1.47	–1.76	–1.79	–1.93	–1.96	–1.73	–1.81	–1.58	–1.58	–1.45	–1.49
^1^E_2_	–1.74	–1.93	2.02	–2.17	–2.23	–1.92	–1.94	–1.74	–1.82	–1.74	–1.76

With an increase of two electrons in the complex due to the second reduction, two possible spin states need to be evaluated: the triplet and the closed-shell singlet. For all reduced solvato complexes, the most stable spin state is the triplet, as shown by the less negative potentials for E_2_. Also, an important difference between the open-shell and closed-shell states is that, for all complexes in the closed-shell singlet state, both reductions occur at the terpyridine ligand.

The oxazole and 1,2,3-triazole rings exhibit electrochemical behavior similar to that of bipyridine, with the second reduction localized on the bidentate ligand, occurring at approximately −1.47 and −1.58 V, respectively. In contrast, **NHC**, pyrrole, and pyrazole show more negative ^3^E_2_ values, which can be attributed to a subsequent reduction centered on the tridentate ligand rather than on the bipyridine fragment. Figure 5 presents the spin-density distribution upon sequential reduction and illustrates representative examples of both behaviors.

**Figure 5:**
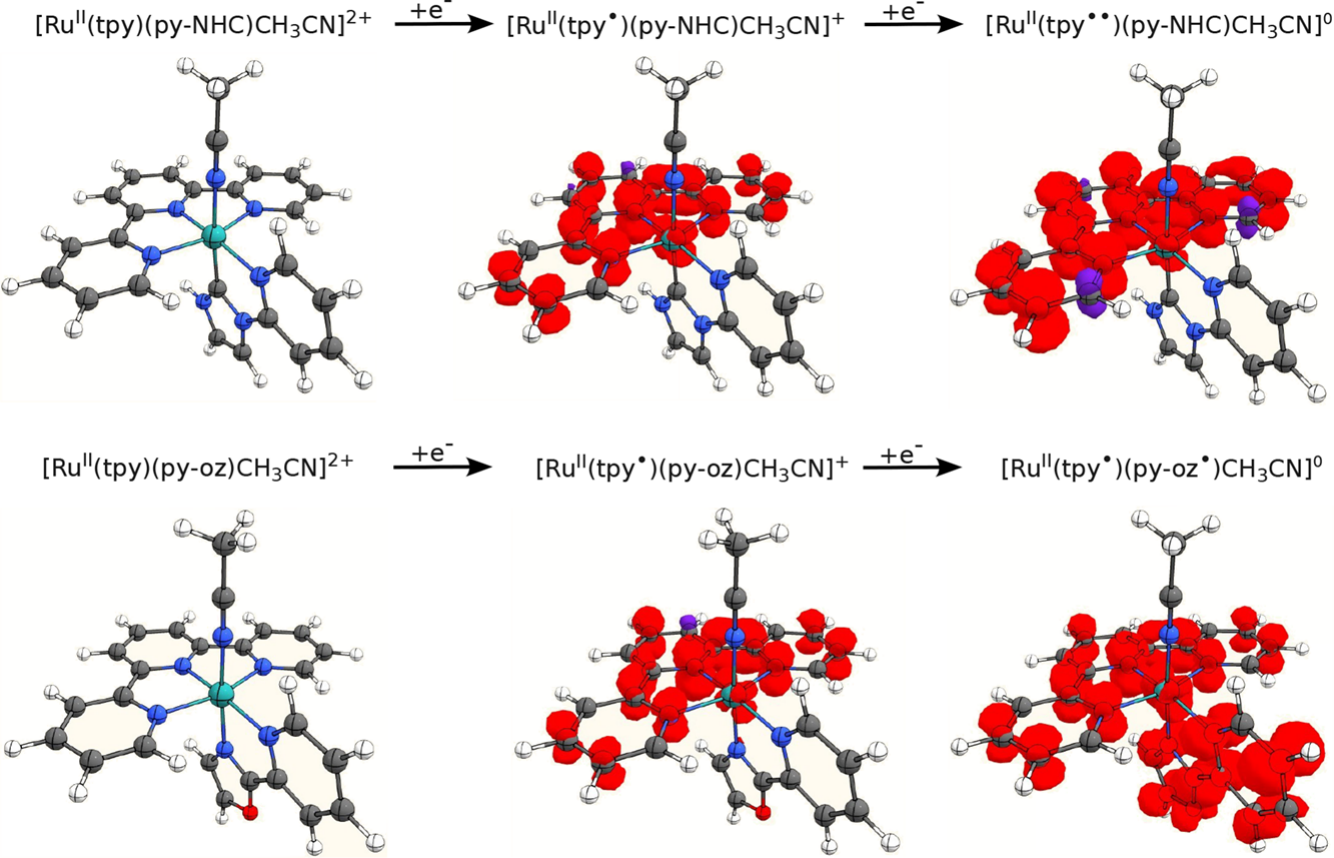
Spin-density plot of sequential reductions for *trans*-[Ru­(tpy)­(py-L)­AcCN]^2+^ with L = **NHC** (top) and **oz** (bottom).

In the benzo-oxazole analogue reported in the literature, the second reduction is experimentally assigned to a double reduction of the terpyridine unit at −1.38 V versus NHE.^20^ However, our theoretical calculations, supported by the spin-density map, clearly show that the added electron is localized on the bidentate oxazole ligand rather than on the terpyridine moiety. Therefore, this provides a more reliable assignment of the second reduction process.

A small difference in reduction potentials was observed when geometric isomers were compared, especially for those groups where terpyridine is doubly reduced. The position of the five-membered ring causes a shift of tens of mV to a lower potential for the *trans* isomer when the pyridine ring is on the opposite side of the central terpyridine Ru–N bond.^44^

When evaluating the dissociation reaction, at ^2^E_1_, the dissociation energies (*D*_E_1__) are considerably larger than those at ^3^E_2_(*D*_E_2__) (Table 2), suggesting that dissociation is more likely to occur in the doubly reduced complex [Ru­(tpy)­(py–L)­(AcCN)]^0^. The highest *D*_E_2__ value was found for the bipyridine and oxazole complexes, with energies of approximately 8 kcal/mol (Table 2). All Δ_T–S_ are positive (Table S1), indicating a forbidden spin-transition reaction, as observed before for the **bpy** complex.

**Table 2:** Dissociation Energies (*D*) in Δ*G* and Dissociation Activation Barrier (Δ*G*_D_^‡^) in kcal/mol for Both Isomers and Distortion Parameter τ_5_ for the Pentacoordinate Complex

		NHC		pyrr		prz		123-trz		oz	
ligands isomers	bpy	*cis*	*trans*	*cis*	trans	*cis*	*trans*	*cis*	*trans*	*cis*	*trans*
D_*E*_1__	14.8	15.5	9.6	15.1	14.9	15.0	15.7	14.7	15.2	15.4	15.9
D_*E*_2__	8.0	–1.4	–1.3	3.4	3.9	1.4	–0.6	3.9	3.0	8.9	7.7
Δ*G*_D_^‡^(E_2_)	17.2	14.8	7.9	16.6	15.9	14.4	12.9	15.9	14.9	19.0	19.0
τ_5_ ^a^	0.43	0.22		0.48		0.35		0.30		0.28	

a τ_5_ values vary from zero to one, where zero defines a perfect square pyramidal geometry and 1.00 is a perfect trigonal bipyramidal.

CV experiments reported the second reduction at −1.31 V versus NHE for the solvato benzo analogue of **NHC** (**MeBim**), which shows a 0.5 V more positive ^3^E_2_ value than that of its model.^18^ However, reduction of *trans*-[Ru­(tpy)­(py–NHC)]^+^ (dissociation product in ^2^E_1_) has a theoretical potential of −1.32 V versus SHE (Table S2) and diverges from the experimental value by only 10 mV, while the optimization attempt of the singlet square pyramidal *cis*-[Ru­(tpy)­(py–NHC)]^0^ always leads to the *trans* position isomer. These potentials suggest that AcCN dissociates before ^3^E_2_ for the **NHC** complex and *cis*-[Ru­(tpy)­(py–NHC)]^+^ isomerizes into the *trans* configuration with Δ*G* = −7.4 kcal/mol, which favors **NHC** *trans* to the vacant position (Figure 6).

**Figure 6:**
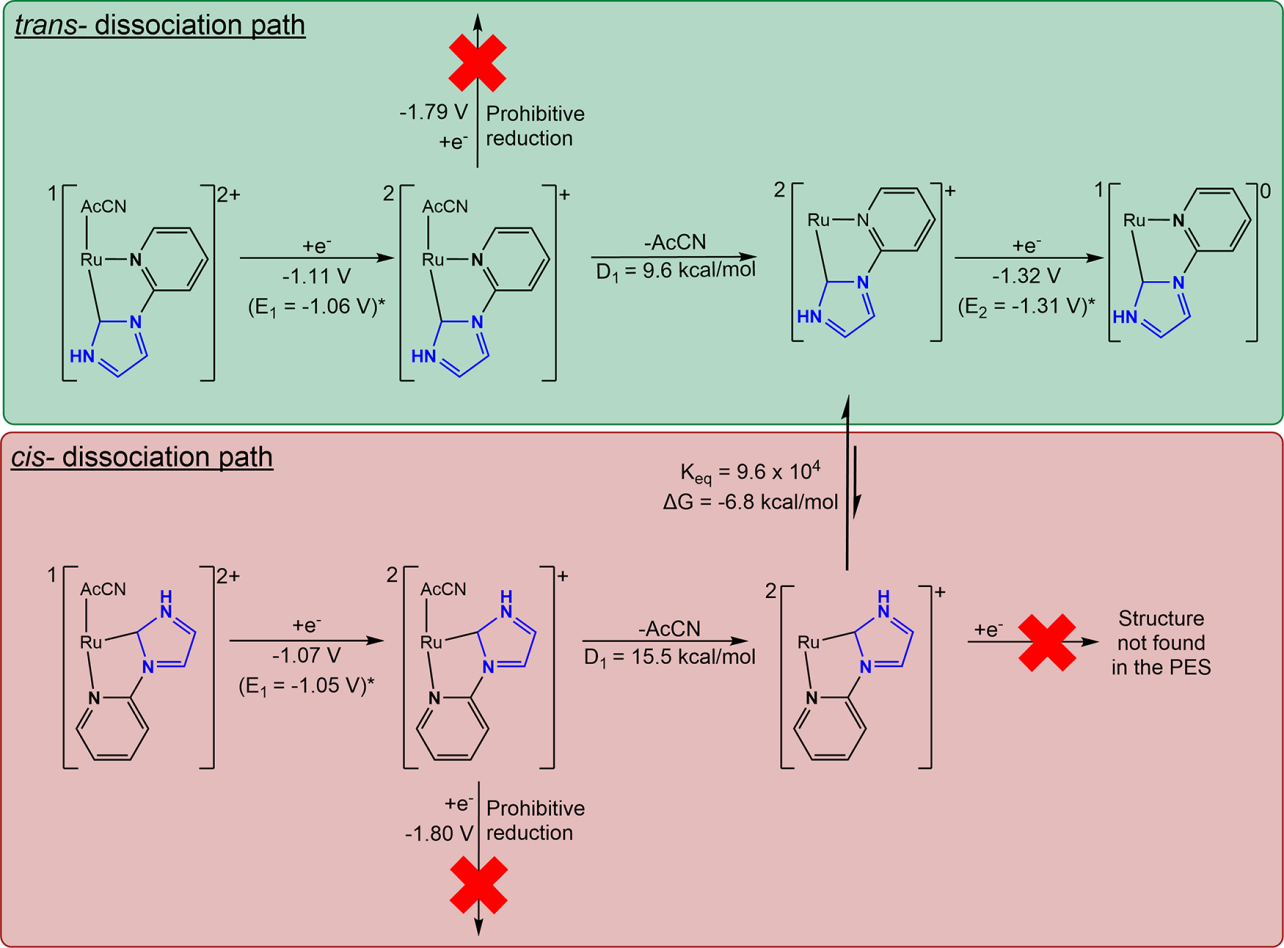
Reaction scheme for the dissociation and isomerization of *trans*- (upper scheme) and *cis*- (lower scheme) **NHC** complexes, where potentials are reported vs NHE, and the values in parentheses are experimental values reported for **MeBim** complexes.^18^

Attempts to find the dissociation TS for ^2^[Ru­(tpy^·^)­(py–NHC)­(AcCN)]^+^ were unsuccessful. However, considering AcCN dissociation from the **NHC** complex at ^2^E_1_ followed by reduction provides a better fit to the experimental report,^18^ and we predict that this pattern will occur for **prz** and **pyrr** since they have similar reduction potentials and are located on the terpyridine ligand.

All transition states of the dissociation step were found in the closed-shell singlet state and showed plausible activation barriers at room temperature.^45^ They are very similar to the structure of the **bpy** ligand and are presented in the Supporting Information. Attempts to obtain dissociation TS in the triplet state for all complexes led to very high gradients (nonconverged structures) compared to the standard convergence parameters.

When comparing *trans* complexes, a *trans*-effect series can be drawn using the Δ*G*_D_^‡^(*E*_2_) values. The ascending order of the ligand series is **oz** < **bpy** < **pyrr** < **123-trz** < **prz** ≪ **NHC**. Also, the smaller *cis*-effect is noticeable and it is not negligible, with **oz** as the only ligand slower than [Ru­(tpy^·^)­(bpy^·^)­AcCN]^0^. The activation barriers for the **NHC** and **prz** ligands are ∼3 kcal/mol smaller than that for **bpy**.

Nagao et al. also described carbon–carbon coupling with the [Ru­(tpy)­(bpy)­CO]^2+^ catalyst at −20 °C for electrocatalysis and stoichiometric reactions with BH_4_^–^. They claimed that the formation of highly reduced CO_2_ products, such as MeOH and C_2_, depends on the thermal lability of Ru–CO dissociation.^12^ This statement suggests that a ligand with a higher dissociation barrier, for example, the 2-pyridine-oxazole­(**oz**), which has a smaller *trans*-effect than **bpy**, could enable the formation of multiple reduction products.

After acetonitrile dissociation, either *cis* or *trans* isomers produce the same pentacoordinated species from the IRC calculations, with most structures closer to square pyramidal (SP) than trigonal bipyramidal (TBP), as identified by the τ_5_ distortion parameter^46^ (Figure 7). The τ_5_ values were obtained by assuming that the angle between terminal pyridines of tpy is 180°, since in either geometry these nitrogen atoms must be in an axial position in TBP or in the equatorial plane in SP geometry, and by using the highest angle N-*Rû*-L between the nitrogen atom of terpyridine’s central ring and the donor atom of the five-membered ring (ϕ_L_) or pyridine­(ϕ_py_).

**Figure 7:**
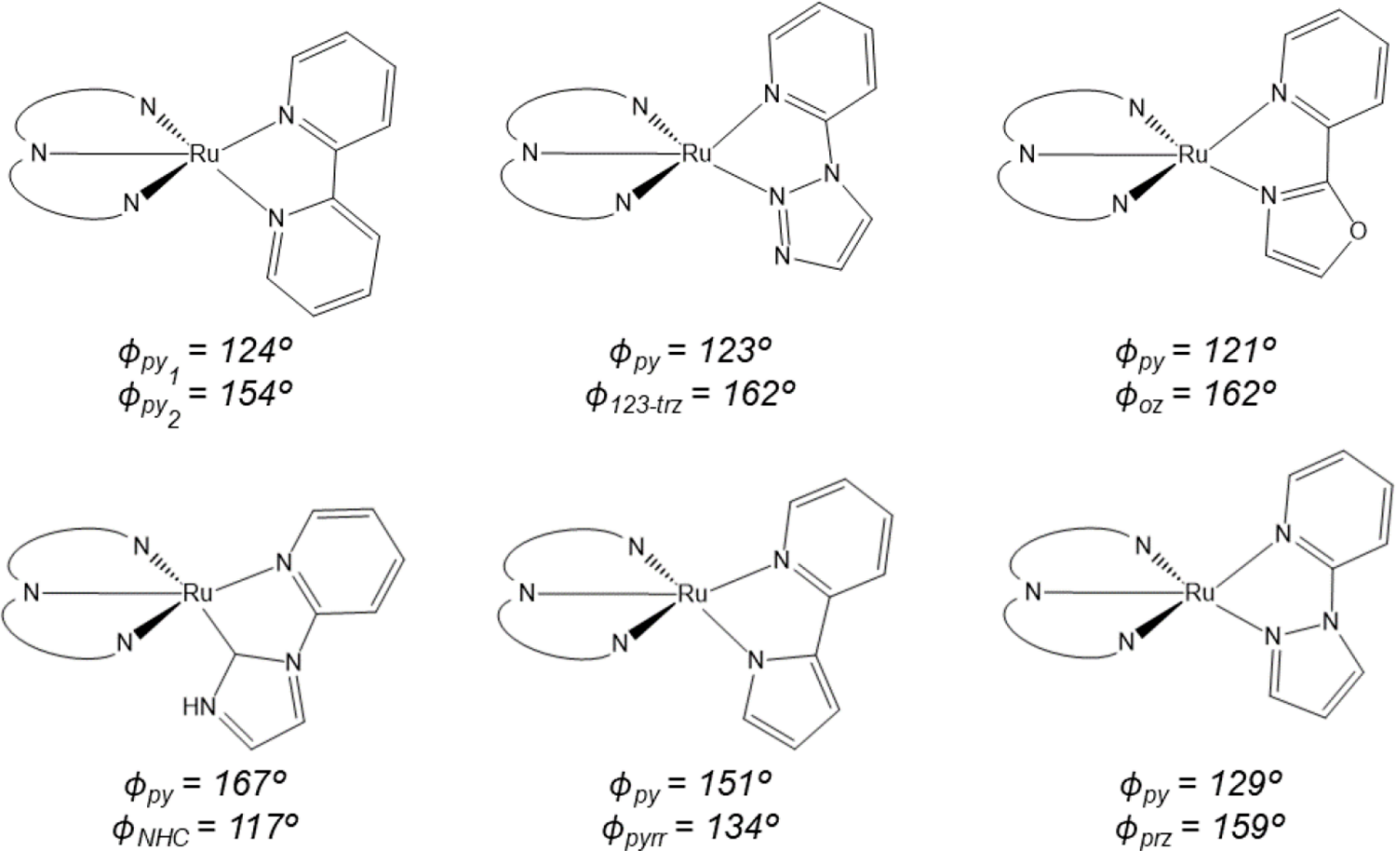
2D structures for the pentacoordinated intermediate [Ru­(tpy)­(py-L)]^0^ with both ϕ_L_ and ϕ_py_.

Calculations of CO_2_ association barriers in Gibbs free energy (Δ*G*_A_^‡^) are presented in Table 3, and the structures are presented in the Supporting Information. The activation barrier indicates a faster reaction than the dissociation step, with Δ*G*_A_^‡^ ≤ 9.0 kcal/mol, consuming the dissociation product and shifting the equilibrium toward the forward reaction, as observed in the microkinetics for the **bpy** complex. The pentacoordinate complex reacts with carbon dioxide, reducing it to afford ^1^[Ru­(tpy)­(py-L)­(CO_2_^··^)]^0^. The transition structure of CO_2_ association occurs via an approximation of the carbon toward ruthenium from the terpyridine side.

**Table 3:** Association Reaction Energies (Δ_R_ *G*_A_) and Activation Barriers (Δ*G*_A_^‡^), in kcal/mol, for Both Isomers, and the *Cis*:*Trans* Proportion of Metallocarboxylate Isomers

		NHC		pyrr		prz		123-trz		oz	
Ligands isomers	bpy	*cis*	*tran*s	*cis*	*trans*	*cis*	*trans*	*cis*	*trans*	*cis*	*trans*
Δ_R_ *G*_A_	–14.6	–16.0	–7.8	–17.6	–17.3	–16.0	–14.8	–13.8	–14.4	–17.7	–16.3
Δ*G*_A_^‡^	5.0	9.0	6.6	7.1	6.5	4.9	5.5	4.3	5.4	3.8	5.4
*cis*:*trans*		1:57.4		1:2.8		2.8:1		6.4:1		14.9:1	

This step can lead to *cis* and *trans* isomers, depending on which terpyridine’s side the CO_2_ associates with the ruthenium center. Gonell et al.^18^ described this isomerization for the complex [Ru­(tpy)­(MeBim-py)­AcCN]^2+^ with **MeBim-py**, where the precatalyst *cis*-[Ru­(tpy)­(MeBim-py)­CO_2_]^0^ is used to reduce CO_2_ and it isomerizes to *trans*-[Ru­(tpy)­(MeBim-py)­CO_2_]^0^. The authors attributed this isomerization to the lower angle of the MeBim-*Rû*-N than the counterpart with pyridine in the pentacoordinate complex. This correlation was also observed when comparing ϕ for the same ligand, and this can be used to qualitatively predict which isomer will form in excess during catalysis.

Microkinetic modeling for the py-L complexes closely resembles that of the **bpy** system, even in the occurrence of competing *cis* and *trans* CO_2_ addition pathways (Figure S4), because the kinetic and thermodynamic products are identical. In contrast, for the **NHC** system, the kinetic and thermodynamic products differ: the *trans* isomer is formed first due to a lower activation barrier, whereas the *cis* isomer is the most thermodynamically stable species (Table 3).

To obtain quantitative information on isomer distribution, the ratio $\frac{\Delta G_{A_{\mathrm{trans}}}^{‡}}{\Delta G_{A_{\mathrm{cis}}}^{‡}}$ was calculated and is presented in Table 3 as *cis*:*trans* proportion. The **NHC** and **pyrr** ligands produce a *trans* excess compared to the other ligands. Plotting the correlation between the isomer excess and the geometric index τ_5_ (Figure 8a) shows how available the ruthenium(0) center is for CO_2_ association from one side rather than the other, i.e., how close the pentacoordinate is to the SP geometry. The exponential correlation between the geometric distortion parameter τ_5_ and *cis*:*trans* proportion (Figure 8) arises from the Eyring equation (eq 4) when $\frac{k_{\mathrm{trans}}}{k_{\mathrm{cis}}}$ is obtained.

**Figure 8:**
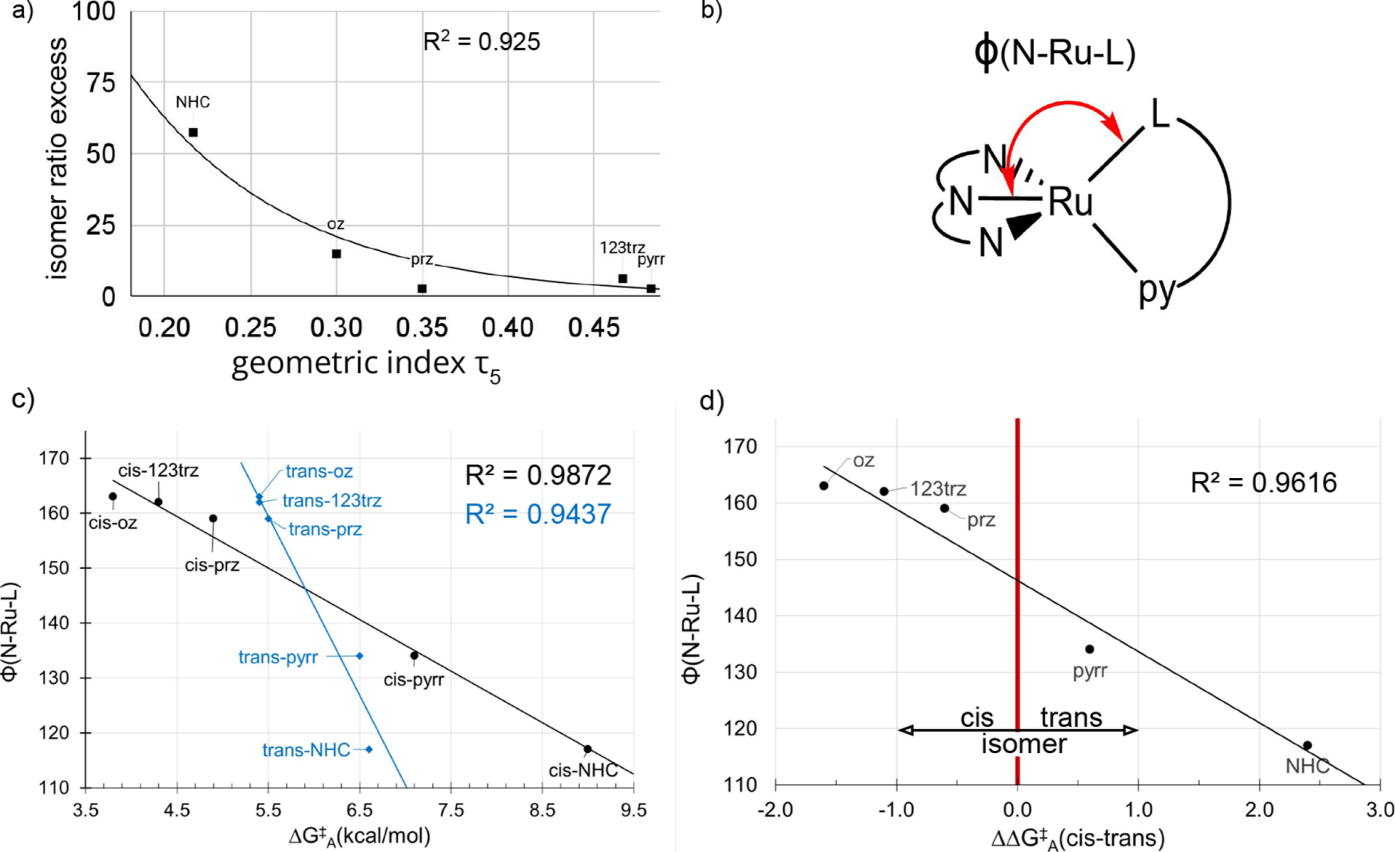
(a) Plot of τ_5_ against excess isomer ratio for the five-membered ring complexes, (b) 2D drawing showing the Φ­(N–Ru-L) angle, (c) plot of ϕ­(N–Ru-py) against Δ*G*_A_^‡^, and (d) plot of ϕ­(N–Ru-L) against ΔΔ*G*_A_^‡^(*cis*–*trans*).

A plot of Φ­(N–Ru–L) (Figure 8b) versus Δ*G*_A_^‡^ (Figure 8c) reveals that higher activation barriers correlate with increased negative charge on the donor atom. Ligands featuring strongly donating atoms, such as the amide nitrogen in pyrrole and the carbene carbon in **NHCs**, exhibit larger Δ*G*_A_^‡^ values compared to ligands containing imine nitrogen donors, which display lower barriers.

For the *cis* series, steric effects dominate the observed trend. In this configuration, the ligand *trans* to the CO_2_ addition site is consistently pyridine, rendering the electronic contribution effectively constant across the bidentate ligands. Consequently, variations in Δ*G*_A_^‡^ primarily reflect steric hindrance. In contrast, for the *trans* series, electronic effects become more pronounced because different donor rings occupy the position *trans* to the reaction site. This variability enhances the electronic influence on the activation barrier, resulting in a steeper slope relative to the *cis* trend. Compared to the *cis* series, this behavior is reflected directly in the *trans* correlation, where **NHC** and pyrrole derivatives occupy the low-Δ*G*_A_^‡^ region, while oxazole, pyrazole, and 1,2,3-triazole cluster at higher activation barriers, indicative of slower *trans* addition.

A second way to correlate the data is to plot Φ­(N–Ru–L) versus ΔΔ*G*_A_^‡^(*cis*–*trans*). This representation leads to the same conclusion as the correlation shown in Figure 8c, but the trend is more clearly visualized. Negative values indicate preferential formation of the *cis* isomer, positive values favor the *trans* isomer, and values close to zero correspond to nonselective formation. The major isomer can be qualitatively determined by identifying the Φ­(N–Ru-L) angle in ^1^[Ru^0^(tpy)­(py-L)]^0^. Also, a higher proportion of one isomer relative to the other can be expected for lower values of τ_5_ for new ligands with similar steric effects.

## Conclusions

In this study, we computationally described the formation of the metallocarboxylated ruthenium complex ^1^[Ru^*II*^(tpy)­(py-L)­(CO_2_^2–^)]^0^ in anhydrous media electrocatalysis for a series of bidentate ligands with rings of 2-pyridine-5 members and well-studied [Ru­(tpy)­(bpy)­S]^2+^. All complexes undergo a slow dissociation step followed by fast association through a pentacoordinate complex, which determines the geometric isomer formed before the catalytic cycle.

All complexes with two reductions on both ligands in open-shell states have less negative potentials than systems in which both electrons are located on terpyridine. However, experimental data in the literature show a lower catalytic potential, suggesting that dissociation occurs at the first reduction for these ligands. Since all first reductions are localized on the terpyridine ligand, modification of the bidentate ligand, such as extending the π system by adding a benzo group to its backbone, lowers the second reduction potential by stabilizing the nonligand π* molecular orbital energy of the bidentate ligand, bringing it closer to that of the terpyridine ligand.

Association activation barriers are necessary to quantitatively discuss isomer distribution instead of only using association thermodynamics for this type of complex. From a kinetic perspective, since the dissociation step activates the precatalyst (^3^[Ru­(tpy)­(py-L)­(AcCN)]^0^) and its backward reaction leads to an off-side species, a ligand with a higher Δ*G*_D_^‡^ (i.e., slower dissociation) may still display a higher TOF than a complex with faster dissociation, provided that this activation/deactivation step is not rate-limiting. To test this hypothesis, the full catalytic cycle for CO_2_ reduction to CO under anhydrous conditions must be evaluated.

Carbon dioxide addition selectivity in Ru(0)-py-L complexes is controlled by geometric distortion and ligand-dependent steric and electronic effects. The *cis* : *trans* ratio follows an exponential dependence on the pentacoordinate distortion parameter τ_5_, consistent with Eyring kinetics. For the small ligand set studied, strongly donating ligands such as **NHC** and pyrrole favor *trans* addition, while weaker donors lead to higher barriers and reduced selectivity. The Φ­(N–Ru–L) angle provides a simple and intuitive descriptor for predicting the preferred isomer and relative selectivity in related systems.

## Supplementary Material







## Notes

Energies obtained from calculations (PDF)
Cartesian coordinates (XYZ) for all structures in this paper (ZIP)
Me-NHC ligand, microkinetics for all ligands, and further computational details (PDF)

## References

[1] Friedlingstein P., O’Sullivan M. W., Jones Mand, Andrew R. M., Gregor L., Hauck J., Le Quéré C., Luijkx I. T., Olsen A., Peters G. P., Peters W., Pongratz J., Schwingshackl C., Sitch S., Canadell J. G., Ciais P., Jackson R. B., Alin S. R., Alkama R., Arneth A., Arora V. K., Bates N. R., Becker M., Bellouin N., Bittig H. C., Bopp L., Chevallier F., Chini L. P., Cronin M., Evans W., Falk S., Feely R. A., Gasser T., Gehlen M., Gkritzalis T., Gloege L., Grassi G., Gruber N., Gürses, Harris I., Hefner M., Houghton R. A., Hurtt G. C., Iida Y., Ilyina T., Jain A. K., Jersild A., Kadono K., Kato E., Kennedy D., Klein Goldewijk K., Knauer J., Korsbakken J. I., Landschützer P., Lefèvre N., Lindsay K., Liu J., Liu Z., Marland G., Mayot N., McGrath M. J., Metzl N., Monacci N. M., Munro D. R., Nakaoka S.-I., Niwa Y., O’Brien K., Ono T., Palmer P. I., Pan N., Pierrot D., Pocock K., Poulter B., Resplandy L., Robertson E., Rödenbeck C., Rodriguez C., Rosan T. M., Schwinger J., Séférian R., Shutler J. D., Skjelvan I., Steinhoff T., Sun Q., Sutton A. J., Sweeney C., Takao S., Tanhua T., Tans P. P., Tian X., Tian H., Tilbrook B., Tsujino H., Tubiello F., van der Werf G. R., Walker A. P., Wanninkhof R., Whitehead C., Willstrand Wranne A., Wright R., Yuan W., Yue C., Yue X., Zaehle S., Zeng J., Zheng B. (2022). Global carbon budget
2022. Earth Syst. Sci. Data Discuss..

[2] Ma M., Trześniewski B. J., Xie J., Smith W. A. (2016). Selective
and efficient reduction of carbon dioxide to carbon monoxide on oxide-derived
nanostructured silver electrocatalysts. Angew.
Chem..

[3] Han N., Ding P., He L., Li Y., Li Y. (2020). Promises of
main group metal–based nanostructured materials for electrochemical
CO2 reduction to formate. Adv. Energy Mater..

[4] Meskine H., Albin V., Cassir M., Ringuedé A., Lair V. (2021). Electrochemical investigations on CO2 reduction mechanism in molten
carbonates in view of H2O/CO2 co-electrolysis. Int. J. Hydrogen Energy.

[5] Fors S. A., Malapit C. A. (2023). Homogeneous Catalysis
for the Conversion of CO2, CO,
CH3OH, and CH4 to C2+ Chemicals via C–C Bond Formation. ACS Catal..

[6] Liu Q., Wu L., Jackstell R., Beller M. (2015). Using carbon dioxide
as a building
block in organic synthesis. Nat. Commun..

[7] Yang H., Zhang C., Gao P., Wang H., Li X., Zhong L., Wei W., Sun Y. (2017). A review of the catalytic
hydrogenation of carbon dioxide into value-added hydrocarbons. Catal. Sci. Technol..

[8] Queyriaux N. (2021). Redox-Active
Ligands in Electroassisted Catalytic H+ and CO2 Reductions: Benefits
and Risks. ACS Catal..

[9] Tanaka K., Ooyama D. (2002). Multi-electron reduction
of CO2 via Ru CO2, C (O) OH,
CO, CHO, and CH2OH species. Coord. Chem. Rev..

[10] Francke R., Schille B., Roemelt M. (2018). Homogeneously
catalyzed electroreduction
of carbon dioxide—methods, mechanisms, and catalysts. Chem. Rev..

[11] Younus H. A., Ahmad N., Ni W., Wang X., Al-Abri M., Zhang Y., Verpoort F., Zhang S. (2023). Molecular
catalysts
for CO2 Electroreduction: Progress and prospects with pincer type
complexes. Coord. Chem. Rev..

[12] Nagao H., Mizukawa T., Tanaka K. (1994). Carbon-Carbon
Bond Formation in the
Electrochemical Reduction of Carbon Dioxide Catalyzed by a Ruthenium
Complex. Inorg. Chem..

[13] Chen Z., Chen C., Weinberg D. R., Kang P., Concepcion J. J., Harrison D. P., Brookhart M. S., Meyer T. J. (2011). Electrocatalytic
reduction of CO2 to CO by polypyridyl ruthenium complexes. Chem. Commun..

[14] Chen Z., Concepcion J. J., Brennaman M. K., Kang P., Norris M. R., Hoertz P. G., Meyer T. J. (2012). Splitting CO2 into CO and O2 by a
single catalyst. Proc. Natl. Acad. Sci. U. S.
A..

[15] Queyriaux N., Esmieu C., Gupta A. K., Vendier L., Ott S., Orio M., Hammarström L. (2021). Electrochemical, Spectroscopic, and
Computational Investigation of a Series of Polypyridyl Ruthenium (II)
Complexes: Characterization of Reduced States. Eur. J. Inorg. Chem..

[16] Johnson B. A., Maji S., Agarwala H., White T. A., Mijangos E., Ott S. (2016). Activating a Low Overpotential CO2 Reduction Mechanism by a Strategic
Ligand Modification on a Ruthenium Polypyridyl Catalyst. Angew. Chem., Int. Ed..

[17] Duan L., Manbeck G. F., Kowalczyk M., Szalda D. J., Muckerman J. T., Himeda Y., Fujita E. (2016). Noninnocent Proton-Responsive Ligand
Facilitates Reductive Deprotonation and Hinders CO2 Reduction Catalysis
in [Ru (tpy)­(6DHBP)­(NCCH3)] 2+(6DHBP= 6, 6-(OH) 2bpy). Inorg. Chem..

[18] Gonell S., Massey M. D., Moseley I. P., Schauer C. K., Muckerman J. T., Miller A. J. (2019). The Trans Effect in Electrocatalytic
CO2 Reduction:
Mechanistic Studies of Asymmetric Ruthenium Pyridyl-Carbene Catalysts. J. Am. Chem. Soc..

[19] Gonell S., Assaf E. A., Duffee K. D., Schauer C. K., Miller A. J. M. (2020). Kinetics
of the Trans Effect in Ruthenium Complexes Provide Insight into the
Factors That Control Activity and Stability in CO2 Electroreduction. J. Am. Chem. Soc..

[20] Chanda N., Paul D., Kar S., Mobin S. M., Datta A., Puranik V. G., Rao K. K., Lahiri G. K. (2005). Effect of 2-(2-Pyridyl)­azole-Based
Ancillary Ligands L1–4 on the Electrophilicity of the Nitrosyl
Function in [RuII­(trpy)­(L1–4)­(NO)]­3+[trpy = 2,2‘:6‘,2“-Terpyridine].
Synthesis, Structures, and Spectroscopic, Electrochemical, and Kinetic
Aspects. Inorg. Chem..

[21] Wu F., Chamchoumis C. M., Thummel R. P. (2000). Bidentate Ligands That Contain Pyrrole
in Place of Pyridine. Inorg. Chem..

[22] Wang Y., Zhang Y., Yang B., Zhang A., Yao Q. (2015). N-(1-Oxy-2-picolyl)­oxalamic
acids as a new type of O,O-ligands for the Cu-catalyzed N-arylation
of azoles with aryl halides in water or organic solvent. Org. Biomol. Chem..

[23] Laemmel A.-C., Collin J.-P., Sauvage J.-P. (2000). Photosubstitution of ancillary ligands
in octahedral mono-terpyridine ruthenium (II) complexes. C. R. Acad. Sci. IIc Chem..

[24] Keith J. M. (2010). One-Step
Conversion of Azine N-Oxides to a-1,2,4-Triazolo-, 1,2,3-Triazolo,
Imidazolo-, and Pyrazoloheteroarenes. Journal
of Organic Chemistry.

[25] Richardson C., Steel P. J. (2003). Benzotriazole as a structural component in chelating
and bridging heterocyclic ligands; ruthenium, palladium, copper and
silver complexes. Dalton Trans..

[26] Neese F. (2012). The ORCA program
system. WIREs Comput. Mol. Sci..

[27] Tao J., Perdew J. P., Staroverov V. N., Scuseria G. E. (2003). Climbing the Density
Functional Ladder: Nonempirical Meta–Generalized Gradient Approximation
Designed for Molecules and Solids. Phys. Rev.
Lett..

[28] Weigend F., Ahlrichs R. (2005). Balanced basis sets
of split valence, triple zeta valence
and quadruple zeta valence quality for H to Rn: Design and assessment
of accuracy. Phys. Chem. Chem. Phys..

[29] Leininger T., Nicklass A., Küchle W., Stoll H., Dolg M., Bergner A. (1996). The accuracy of the
pseudopotential approximation:
non-frozen-core effects for spectroscopic constants of alkali fluorides
XF (X = K, Rb, Cs). Chem. Phys. Lett..

[30] Weigend F. (2006). Accurate Coulomb-fitting
basis sets for H to Rn. Phys. Chem. Chem. Phys..

[31] Vennelakanti V., Taylor M. G., Nandy A., Duan C., Kulik H. J. (2023). Assessing
the performance of approximate density functional theory on 95 experimentally
characterized Fe­(II) spin crossover complexes. J. Chem. Phys..

[32] Weymuth T., Couzijn E. P. A., Chen P., Reiher M. (2014). New Benchmark Set of
Transition-Metal Coordination Reactions for the Assessment of Density
Functionals. J. Chem. Theory Comput..

[33] Grimme S., Antony J., Ehrlich S., Krieg H. (2010). A consistent and accurate
ab initio parametrization of density functional dispersion correction
(DFT-D) for the 94 elements H-Pu. J. Chem. Phys..

[34] Grimme S., Ehrlich S., Goerigk L. (2011). Effect of the damping function in
dispersion corrected density functional theory. J. Comput. Chem..

[35] Tomasi J., Mennucci B., Cammi R. (2005). Quantum mechanical continuum solvation
models. Chem. Rev..

[36] Harvey J. N., Himo F., Maseras F., Perrin L. (2019). Scope and Challenge
of Computational Methods for Studying Mechanism and Reactivity in
Homogeneous Catalysis. ACS Catal..

[37] Hoops S., Sahle S., Gauges R., Lee C., Pahle J., Simus N., Singhal M., Xu L., Mendes P., Kummer U. (2006). COPASI—a COmplex PAthway SImulator. Bioinformatics.

[38] Bartmess J. E. (1994). Thermodynamics
of the Electron and the Proton. J. Phys. Chem..

[39] Harvey J. N., Poli R., Smith K. M. (2003). Understanding
the reactivity of transition
metal complexes involving multiple spin states. Coord. Chem. Rev..

[40] Xie Y., Shaffer D. W., Lewandowska-Andralojc A., Szalda D. J., Concepcion J. J. (2016). Water Oxidation by Ruthenium Complexes Incorporating
Multifunctional Bipyridyl Diphosphonate Ligands. Angew. Chem., Int. Ed..

[41] Fukuda R., Ehara M., Nakatsuji H., Kishimoto N., Ohno K. (2010). Valence ionized states of iron pentacarbonyl and eta-5-cyclopentadienyl
cobalt dicarbonyl studied by symmetry-adapted cluster-configuration
interaction calculation and collision-energy resolved Penning ionization
electron spectroscopy. J. Chem. Phys..

[42] Hiraki K., Kira S.-i., Kawano H. (1997). Formation of Di- and Tricarbonylruthenium­(O)
Species from [RuH2­(CO)­(PPh3)­3] via Decarbonylation of Methyl Benzoates:
X-Ray Crystal Structures of [Ru­(CO)­2­(PPh3)­3] and [Ru­(O2)­(CO)­2­(PPh3)­2]. Bull. Chem. Soc. Jpn..

[43] Bender B. R., Hembre R. T., Norton J. R., Burnell E. E. (1998). Ethylene Ligand
Structures of Os­(CO)­4­(C2H4) and Os2­(CO)­8­(C2H4) Determined by 1H NMR
in Liquid Crystal Solvents. Inorg. Chem..

[44] Lever A. (1991). Electrochemical
parametrization of rhenium redox couples. Inorganic
chemistry.

[45] Ryu H., Park J., Kim H. K., Park J. Y., Kim S.-T., Baik M.-H. (2018). Pitfalls
in Computational Modeling of Chemical Reactions
and How To Avoid Them. Organometallics.

[46] Addison A. W., Rao T. N., Reedijk J., van Rijn J., Verschoor G. C. (1984). Synthesis,
structure, and spectroscopic properties of copper­(II) compounds containing
nitrogen–sulphur donor ligands; the crystal and molecular structure
of aqua­[1,7-bis­(N-methylbenzimidazol-2’-yl)-2,6-dithiaheptane]­copper­(II)
perchlorate. J. Chem. Soc., Dalton Trans..

