# Supplementary material for: The Effect of N‑Donor Ligands on Activation of [Ru(tpy)(L–py)(AcCN)]2+ Precatalysts in CO2 Reduction Reaction Electrocatalysis
Source: Inorg Chem. 2026 Jun 18;65(26):15164–73. doi: 10.1021/acs.inorgchem.6c02082 (PMC13343509; doi:10.1021/acs.inorgchem.6c02082)
Supplement: Supplementary file 1 [file ic6c02082_si_001.pdf]

BPY

					E(TZVP)+(Esvp - Gsvp)
	Pontial de2-TZVP	Potential def2-SVP (Eh)	G (Eh)	Gcorr (kcal/mol)	E + Gcorr (kcal/mol)
^{1}[Ru(tpy)(bpy)AcCN]$^{2+}$	-1466.12777687	-1464.67487725	-1464.28789093	-918852.67229354	-919764.38060743
^{2}[Ru(tpy)(bpy)AcCN]$^{+}$	-1466.23948287	-1464.78680136	-1464.40708258	-918927.46618624	-919839.03763578
^{1}[Ru(tpy)(bpy)AcCN]^{0}	-1466.33016771	-1464.87669773	-1464.49911076	-918985.21474345	-919897.28095876
^{3}[Ru(tpy)(bpy)AcCN]^{0}	-1466.33678013	-1464.88372201	-1464.50961302	-918991.80501137	-919903.61278228
^{2}[Ru(tpy)(bpy)]$^{+}$	-1333.37468997	-1332.06498415	-1331.72534178	-835668.41335770	-836490.26619903
^{1}[Ru(tpy)(bpy)]^{0}	-1333.48555424	-1332.17449058	-1331.83779311	-835738.97763556	-836561.68254032
^{3}[Ru(tpy)(bpy)]^{0}	-1333.47288591	-1332.16324289	-1331.82888657	-835733.38869710	-836555.20213370
^{1}[Ru(tpy)(bpy)CO_2]^{0}	-1522.20000703	-1520.66448451	-1520.31468510	-954010.01788976	-954973.57286148
^{3}[Ru(tpy)(bpy)CO_2]^{0}	-1522.16454813	-1520.63447225	-1520.29086760	-953995.07218224	-954955.20933368
^{3}[TS Associative]^{0}	-1654.99035637	-1653.31513100	-1652.92984471	-1037227.29038905	-1038278.51021892
TS Dissociation	-1466.31162140	-1464.85557916	-1464.47927874	-918972.76996250	-919886.45029992
TS Association	-1522.16449841	-1520.63454367	-1520.28909068	-953993.95714806	-954954.01828434

NHC

CIS					E(TZVP)+(Esvp - Gsvp)
	Pontial de2-TZVP	Potential energy (Eh)	G (Eh)	Gcorr (kcal/mol)	E + Gcorr (kcal/mol)
^{1}[Ru(tpy)(py-NHC)AcCN]$^{2+}$	-1444.03490745	-1442.60034500	-1442.23081677	-905011.64871593	-905911.85028058
^{2}[Ru(tpy)(py-NHC)AcCN]$^{+}$	-1444.14570775	-1442.71109821	-1442.34834818	-905085.40079226	-905985.63191032
^{1}[Ru(tpy)(py-NHC)AcCN]^{0}	-1444.22723737	-1442.79009712	-1442.43170684	-905137.70914332	-906039.52830073
^{3}[Ru(tpy)(py-NHC)AcCN]^{0}	-1444.23467752	-1442.79889090	-1442.43929316	-905142.46963119	-906043.43937306
^{2}[Ru(tpy)(py-NHC)]$^{+}$	-1311.27967963	-1309.98801527	-1309.66550062	-821825.65346131	-822636.18511795
^{1}[Ru(tpy)(py-NHC)]^{0}	-1311.39603898	-1310.10300541	-1309.78320373	-821899.51328101	-822710.90413322
^{3}[Ru(tpy)(py-NHC)]^{0}	-1311.37077345	-1310.07865868	-1309.76156340	-821885.93376835	-822696.74806146
^{1}[Ru(tpy)(py-NHC)CO_2]^{0}	-1500.11087823	-1498.59364183	-1498.26257348	-940172.10835315	-941124.18860968
TS Dissociation	-1444.21166746	-1442.77348031	-1442.41337300	-905126.20448454	-906028.68058350
TS Association	-1500.06752638	-1498.55551509	-1498.22800388	-940150.41560074	-941099.21704834

TRANS					E(TZVP)+(Esvp - Gsvp)
	Pontial de2-TZVP	Potential energy (Eh)	G (Eh)	Gcorr (kcal/mol)	E + Gcorr (kcal/mol)
^{1}[Ru(tpy)(py-NHC)AcCN]$^{2+}$	-1444.03716581	-1442.60230807	-1442.23336708	-905013.24905969	-905913.63591947
^{2}[Ru(tpy)(py-NHC)AcCN]$^{+}$	-1444.14593166	-1442.71092194	-1442.34942012	-905086.07344479	-905986.55567678
^{1}[Ru(tpy)(py-NHC)AcCN]^{0}	-1444.22518941	-1442.78748148	-1442.42915081	-905136.10521021	-906038.28059032
^{3}[Ru(tpy)(py-NHC)AcCN]^{0}	-1444.23403165	-1442.79774049	-1442.43893668	-905142.24593660	-906043.53228325
^{2}[Ru(tpy)(py-NHC)]$^{+}$	-1311.29036615	-1309.99900021	-1309.67669037	-821832.67513573	-822643.01953420
^{1}[Ru(tpy)(py-NHC)]^{0}	-1311.39603898	-1310.10300541	-1309.78320373	-821899.51328101	-822710.90413322
^{3}[Ru(tpy)(py-NHC)]^{0}	-1311.38165485	-1310.08949402	-1309.77209837	-821892.54456211	-822703.38776095
^{1}[Ru(tpy)(py-NHC)CO_2]^{0}	-1500.09918661	-1498.58197628	-1498.24949747	-940163.90303265	-941115.96692791

TS Dissociation	-1444.22224547	-1442.78487413	-1442.42526987	-905133.66988349	-906035.63405350
TS Association	-1500.07176503	-1498.55998534	-1498.23194950	-940152.89151477	-941101.54763677

PYRR

CIS					E(TZVP)+(Esvp - Gsvp)
	Pontial de2-TZVP	Potential energy (Eh)	G (Eh)	Gcorr (kcal/mol)	E + Gcorr (kcal/mol)
^{1}[Ru(tpy)(py-pyrr)AcCN]$^{+}$	-1427.52779533	-1426.11439226	-1425.74732036	-894668.09812544	-895555.02197614
^{2}[Ru(tpy)(py-pyrr)AcCN]^{0}	-1427.63020896	-1426.21655257	-1425.85654702	-894736.63889225	-895623.72170977
^{1}[Ru(tpy)(py-pyrr)AcCN^{-}	-1427.70342364	-1426.28697019	-1425.93049889	-894783.04439322	-895671.88239442
^{3}[Ru(tpy)(py-pyrr)AcCN]$^{-}$	-1427.71268876	-1426.29748720	-1425.94065806	-894789.41936890	-895677.47179187
^{2}[Ru(tpy)(py-pyrr)]^{0}	-1294.76506085	-1293.49478148	-1293.17485293	-811477.61537468	-812274.72775020
^{1}[Ru(tpy)(py-pyrr)]$^{-}$	-1294.86685712	-1293.59436123	-1293.27687525	-811541.63534969	-812340.13860687
^{3}[Ru(tpy)(py-pyrr)]$^{-}$	-1294.84876497	-1293.57727650	-1293.26247665	-811532.60009140	-812330.47118559
^{1}[Ru(tpy)(py-pyrr)CO_2]$^{-}$	-1483.58504933	-1482.08642359	-1481.75694216	-929814.66789635	-930755.06978452
[TS Dissociation]$^{-}$	-1427.68685058	-1426.26909928	-1425.91170705	-894771.25233509	-895660.90474298
[TS Association]$^{-}$	-1483.54198845	-1482.05007165	-1481.72425560	-929794.15676943	-930730.34873269

TRANS					E(TZVP)+(Esvp - Gsvp)
	Pontial de2-TZVP	Potential energy (Eh)	G (Eh)	Gcorr (kcal/mol)	E + Gcorr (kcal/mol)
^{1}[Ru(tpy)(py-pyrr)AcCN]$^{+}$	-1427.52965440	-1426.11612566	-1425.74902550	-894669.16811699	-895556.17083396
^{2}[Ru(tpy)(py-pyrr)AcCN]^{0}	-1427.63172778	-1426.21790407	-1425.85823458	-894737.69785218	-895624.88566263
^{1}[Ru(tpy)(py-pyrr)AcCN^{-}	-1427.70399689	-1426.28751455	-1425.93009973	-894782.79391652	-895671.65004044
^{3}[Ru(tpy)(py-pyrr)AcCN]$^{-}$	-1427.71360046	-1426.29818657	-1425.94118211	-894789.74821526	-895677.93387990
^{2}[Ru(tpy)(py-pyrr)]^{0}	-1294.76717372	-1293.49685667	-1293.17685782	-811478.87346220	-812276.00948379
^{1}[Ru(tpy)(py-pyrr)]$^{-}$	-1294.86685712	-1293.59436123	-1293.27687525	-811541.63534969	-812340.13860687
^{3}[Ru(tpy)(py-pyrr)]$^{-}$	-1294.84970469	-1293.57808861	-1293.26302090	-811532.94161345	-812330.89278441
^{1}[Ru(tpy)(py-pyrr)CO_2]$^{-}$	-1483.58493500	-1482.08686065	-1481.75690535	-929814.64479773	-930754.70068299

[TS Dissociation]$^{-}$	-1427.68917277	-1426.27170221	-1425.91379479	-894772.56241178	-895662.03865420
[TS Association]$^{-}$	-1483.54301247	-1482.05128257	-1481.72529044	-929794.80614136	-930730.88082797

PRZ

CIS					E(TZVP)+(Esvp - Gsvp)
	Pontial de2-TZVP	Potential energy (Eh)	G (Eh)	Gcorr (kcal/mol)	E + Gcorr (kcal/mol)
^{1}[Ru(tpy)(py-prz)AcCN]$^{2+}$	-1444.02156829	-1442.58731059	-1442.21790560	-905003.54683410	-905903.55717096
^{2}[Ru(tpy)(py-prz)AcCN]$^{+}$	-1444.13337870	-1442.69936469	-1442.33673784	-905078.11519361	-905977.97260765
^{1}[Ru(tpy)(py-prz)AcCN]^{0}	-1444.21599251	-1442.77983514	-1442.42089052	-905130.92179976	-906032.12419385
^{3}[Ru(tpy)(py-prz)AcCN]^{0}	-1444.22302853	-1442.78802779	-1442.42903708	-905136.03384355	-906036.51044155
^{2}[Ru(tpy)(py-prz)]$^{+}$	-1311.26812768	-1309.97708030	-1309.65473560	-821818.89830899	-822629.04280312
^{1}[Ru(tpy)(py-prz)]^{0}	-1311.38003160	-1310.08757126	-1309.76826536	-821890.13931192	-822701.17045539
^{3}[Ru(tpy)(py-prz)]^{0}	-1311.35989091	-1310.06860825	-1309.75197561	-821879.91733904	-822690.20947272
^{1}[Ru(tpy)(py-prz)CO_2]^{0}	-1500.09634436	-1498.57883324	-1498.24683537	-940162.23253961	-941114.48518263
TS Dissociation	-1444.20092515	-1442.76381314	-1442.40402688	-905120.33970546	-906022.14114586
TS Association	-1500.05844850	-1498.54732286	-1498.21988674	-940145.32201827	-941093.56771288

TRANS					E(TZVP)+(Esvp - Gsvp)
	Pontial de2-TZVP	Potential energy (Eh)	G (Eh)	Gcorr (kcal/mol)	E + Gcorr (kcal/mol)
^{1}[Ru(tpy)(py-prz)AcCN]$^{2+}$	-1444.02173606	-1442.58740411	-1442.21833090	-905003.81371389	-905903.87063826
^{2}[Ru(tpy)(py-prz)AcCN]$^{+}$	-1444.13302120	-1442.69888390	-1442.33655206	-905077.99861490	-905977.93339163
^{1}[Ru(tpy)(py-prz)AcCN]^{0}	-1444.21479984	-1442.77859123	-1442.41986829	-905130.28034072	-906031.51488800
^{3}[Ru(tpy)(py-prz)AcCN]^{0}	-1444.21977888	-1442.78685514	-1442.42794584	-905135.34908009	-906034.52233938
^{2}[Ru(tpy)(py-prz)]$^{+}$	-1311.26672494	-1309.97570485	-1309.65359894	-821818.18504404	-822628.31241775
^{1}[Ru(tpy)(py-prz)]^{0}	-1311.38003160	-1310.08757126	-1309.76826536	-821890.13931192	-822701.17045539
^{3}[Ru(tpy)(py-prz)]^{0}	-1311.35774609	-1310.06616607	-1309.74957642	-821878.41182453	-822688.89055650
^{1}[Ru(tpy)(py-prz)CO_2]^{0}	-1500.09480979	-1498.57776607	-1498.24541559	-940161.34161417	-941113.30095843

TS Dissociation	-1444.19986392	-1442.76300038	-1442.40340103	-905119.94697864	-906021.59250033
TS Association	-1500.05802424	-1498.54632848	-1498.21827110	-940144.30818883	-941092.91163767

1,2,3-trz

CIS					E(TZVP)+(Esvp - Gsvp)
	Pontial de2-TZVP	Potential energy (Eh)	G (Eh)	Gcorr (kcal/mol)	E + Gcorr (kcal/mol)
^{1}[Ru(tpy)(py-123trz)AcCN]$^{2+}$	-1460.04860030	-1458.59166012	-1458.23399849	-915053.79727546	-915968.04108044
^{2}[Ru(tpy)(py-123trz)AcCN]$^{+}$	-1460.16297114	-1458.70627237	-1458.35514237	-915129.81621103	-916043.90852586
^{1}[Ru(tpy)(py-123trz)AcCN]^{0}	-1460.25067961	-1458.79820705	-1458.45216429	-915190.69838754	-916102.13871715
^{3}[Ru(tpy)(py-123trz)AcCN]^{0}	-1460.25613165	-1458.79951594	-1458.45390871	-915191.79302766	-916105.83322513
^{2}[Ru(tpy)(py-123trz)]$^{+}$	-1327.29801217	-1325.98422588	-1325.67362676	-831870.90469135	-832695.31806783
^{1}[Ru(tpy)(py-123trz)]^{0}	-1327.41181454	-1326.09698539	-1325.78838357	-831942.91567982	-832767.98346322
^{3}[Ru(tpy)(py-123trz)]^{0}	-1327.39286003	-1326.07947569	-1325.77510986	-831934.58630069	-832758.74744910
^{1}[Ru(tpy)(py-123trz)CO_2]^{0}	-1516.12314367	-1514.58171319	-1514.26187681	-950211.82318611	-951179.08546063
TS Dissociation	-1460.23237893	-1458.77270570	-1458.42555257	-915173.99928043	-916089.95810027
TS Association	-1516.09091434	-1514.55640927	-1514.23992517	-950198.04832346	-951160.96482985

TRANS					E(TZVP)+(Esvp - Gsvp)
	Pontial de2-TZVP	Potential energy (Eh)	G (Eh)	Gcorr (kcal/mol)	E + Gcorr (kcal/mol)
^{1}[Ru(tpy)(py-123trz)AcCN]$^{2+}$	-1460.04817449	-1458.59140356	-1458.23445448	-915054.08341352	-915968.22101668
^{2}[Ru(tpy)(py-123trz)AcCN]$^{+}$	-1460.16103066	-1458.70432994	-1458.35387060	-915129.01816327	-916043.11170476
^{1}[Ru(tpy)(py-123trz)AcCN]^{0}	-1460.24769855	-1458.79013824	-1458.44268344	-915184.74906409	-916099.38200827
^{3}[Ru(tpy)(py-123trz)AcCN]^{0}	-1460.25385879	-1458.79713100	-1458.45239519	-915190.84327948	-916104.95380673
^{2}[Ru(tpy)(py-123trz)]$^{+}$	-1327.29582295	-1325.98209332	-1325.67161902	-831869.64481543	-832694.02263835
^{1}[Ru(tpy)(py-123trz)]^{0}	-1327.41181454	-1326.09698539	-1325.78838357	-831942.91567982	-832767.98346322
^{3}[Ru(tpy)(py-123trz)]^{0}	-1327.39023923	-1326.07670125	-1325.77270239	-831933.07559040	-832757.33315022
^{1}[Ru(tpy)(py-123trz)CO_2]^{0}	-1516.12454681	-1514.58491209	-1514.26458561	-950213.52298384	-951179.65839819

TS Dissociation	-1460.23321246	-1458.77390635	-1458.42599791	-915174.27873551	-916090.00718701
TS Association	-1516.08927767	-1514.55521520	-1514.23862934	-950197.23517783	-951159.87395198

OZ					
**CIS**					**E(TZVP)+(Esvp - Gsvp)**
	Pontial de2-TZVP	Potential energy (Eh)	G (Eh)	Gcorr (kcal/mol)	E + Gcorr (kcal/mol)
^{1}[Ru(tpy)(py-oz)AcCN]$^{2+}$	-1463.90115521	-1462.43849344	-1462.08127596	-917468.00043702	-918385.83459428
^{2}[Ru(tpy)(py-oz)AcCN]$^{+}$	-1464.01332373	-1462.55093328	-1462.20092758	-917543.08296526	-918460.74686181
^{1}[Ru(tpy)(py-oz)AcCN]^{0}	-1464.10362763	-1462.64019355	-1462.29251435	-917600.55453351	-918518.87332270
^{3}[Ru(tpy)(py-oz)AcCN]^{0}	-1464.11127983	-1462.64816427	-1462.30378588	-917607.62752567	-918525.74644162
^{2}[Ru(tpy)(py-oz)]$^{+}$	-1331.14785576	-1329.82851770	-1329.51843992	-834283.56147498	-835111.45864563
^{1}[Ru(tpy)(py-oz)]^{0}	-1331.25896760	-1329.93839442	-1329.63104311	-834354.22104644	-835182.89326275
^{3}[Ru(tpy)(py-oz)]^{0}	-1331.24647908	-1329.92696174	-1329.62223562	-834348.69426279	-835176.70392555
^{1}[Ru(tpy)(py-oz)CO_2]^{0}	-1519.97756670	-1518.43156504	-1518.11184846	-952627.71697121	-953597.84770107
TS Dissociation	-1464.08417048	-1462.61848552	-1462.27090402	-917586.99384614	-918506.72508624
TS Association	-1519.93846749	-1518.39858542	-1518.08372657	-952610.07021808	-953576.36084169
**TRANS**					**E(TZVP)+(Esvp - Gsvp)**
	Pontial de2-TZVP	Potential energy (Eh)	G (Eh)	Gcorr (kcal/mol)	E + Gcorr (kcal/mol)
^{1}[Ru(tpy)(py-oz)AcCN]$^{2+}$	-1463.90133510	-1462.43856410	-1462.08152978	-917468.15971148	-918386.06241224
^{2}[Ru(tpy)(py-oz)AcCN]$^{+}$	-1464.01295524	-1462.55039958	-1462.20042220	-917542.76583451	-918460.53340336
^{1}[Ru(tpy)(py-oz)AcCN]^{0}	-1464.10295011	-1462.63937618	-1462.29153820	-917599.94199011	-918518.34853375
^{3}[Ru(tpy)(py-oz)AcCN]^{0}	-1464.11074668	-1462.64751641	-1462.30172689	-917606.33548988	-918524.52638204
^{2}[Ru(tpy)(py-oz)]$^{+}$	-1331.14655400	-1329.82715399	-1329.51714176	-834282.74686725	-835110.68290775
^{1}[Ru(tpy)(py-oz)]^{0}	-1331.25896760	-1329.93839442	-1329.63104311	-834354.22104644	-835182.89326275
^{3}[Ru(tpy)(py-oz)]^{0}	-1331.24449585	-1329.92480583	-1329.62025293	-834347.45010598	-835175.56813410
^{1}[Ru(tpy)(py-oz)CO_2]^{0}	-1519.97570635	-1518.43020462	-1518.11012175	-952626.63344428	-953596.45046218

TS Dissociation	-1464.08249138	-1462.61716850	-1462.26929877	-917585.98653651	-918505.49056141
TS Association	-1519.93731398	-1518.39767788	-1518.08143750	-952608.63380491	-953574.77008684

Me-NHC

CIS					E(TZVP)+(Esvp - Gsvp)
	Pontial de2-TZVP	Potential energy (Eh)	G (Eh)	Gcorr (kcal/mol)	E + Gcorr (kcal/mol)
^{1}[Ru(tpy)(py-Me-NHC)AcCN]$^{2+}$	-1483.36486221	-1481.89095606303	-1481.49531295	-929650.49308160	-930575.38319155
^{2}[Ru(tpy)(py-Me-NHC)AcCN]$^{+}$	-1483.47583180	-1482.00192124161	-1481.61301504	-929724.35226124	-930649.24514090
^{1}[Ru(tpy)(py-Me-NHC)AcCN]^{0}	-1483.55748420	-1482.08113731979	-1481.69567494	-929776.22213376	-930702.64382726
^{3}[Ru(tpy)(py-Me-NHC)AcCN]^{0}	-1483.56483806	-1482.08975757555	-1481.70375622	-929781.29321373	-930706.92023120
^{2}[Ru(tpy)(py-Me-NHC)]$^{+}$	-1350.61044092	-1349.27937883885	-1348.93148100	-846465.42917657	-847300.68327860
^{1}[Ru(tpy)(py-Me-NHC)]^{0}	-1350.72807452	-1349.39575986224	-1349.05028150	-846539.97761892	-847376.01772612
^{3}[Ru(tpy)(py-Me-NHC)]^{0}	-1350.70142157	-1349.36991670181	-1349.02723383	-846525.51498705	-847361.04694131
^{1}[Ru(tpy)(py-Me-NHC)CO_2]^{0}	-1539.43263132	-1537.87578532977	-1537.51700147	-964804.63483394	-965781.57047987
TS Dissociation	-1483.54239674	-1482.06517230060	-1481.67930978	-929765.95284039	-930692.92520962
TS Association	-1539.39957680	-1537.84838911766	-1537.49392193	-964790.15220333	-965763.53721083

TRANS					E(TZVP)+(Esvp - Gsvp)
	Pontial de2-TZVP	Potential energy (Eh)	G (Eh)	Gcorr (kcal/mol)	E + Gcorr (kcal/mol)
^{1}[Ru(tpy)(py-Me-NHC)AcCN]$^{2+}$	-1483.36722393	-1481.89332126699	-1481.49842203	-929652.44405883	-930577.33197965
^{2}[Ru(tpy)(py-Me-NHC)AcCN]$^{+}$	-1483.47615092	-1482.00218151506	-1481.61440016	-929725.22143720	-930650.15124193
^{1}[Ru(tpy)(py-Me-NHC)AcCN]^{0}	-1483.55612701	-1482.07972883804	-1481.69406674	-929775.21297298	-930701.66685344
^{3}[Ru(tpy)(py-Me-NHC)AcCN]^{0}	-1483.56504078	-1482.08980972871	-1481.70451287	-929781.76801880	-930707.48951606
^{2}[Ru(tpy)(py-Me-NHC)]$^{+}$	-1350.62185363	-1349.29132665605	-1348.94318997	-846472.77666648	-847307.69498161
^{1}[Ru(tpy)(py-Me-NHC)]^{0}	-1350.72807452	-1349.39591769535	-1349.05036151	-846540.02782596	-847375.96889138
^{3}[Ru(tpy)(py-Me-NHC)]^{0}	-1350.71340333	-1349.38226023818	-1349.03860131	-846532.64818874	-847367.95312262
^{1}[Ru(tpy)(py-Me-NHC)CO_2]^{0}	-1539.43060725	-1537.87461958094	-1537.51675247	-964804.47858407	-965780.87562814

TS Dissociation	-1483.55359637	-1482.07740834571	-1481.69118002	-929773.40152876	-930699.72353938
TS Association	-1539.40664169	-1537.85541162394	-1537.50173815	-964795.05695564	-965768.46855993

	Pontial de2-TZVP	Potential energy (Eh)	G (Eh)	Gcorr (kcal/mol)	E + Gcorr (kcal/mol)
AcCN	-132.82506875	-132.67528435	-132.65429047	-83239.93748568	-83333.92862394
CO2	-188.67196201	-188.44929740	-188.45842971	-118257.56499811	-118397.28915262

Constants	
RT (kcal/mol)	0.5925
kbT/h (1/s)	6.21E+12
h (J/s)	6.63E-34
kb (J/K)	1.38E-23
T (K)	298.15
R (kcal/mol.K)	1.987E-03
Na	6.022E+23
electron (kcal/mol)	0.867
