# Supplementary material for: The Effect of N‑Donor Ligands on Activation of [Ru(tpy)(L–py)(AcCN)]2+ Precatalysts in CO2 Reduction Reaction Electrocatalysis
Source: Inorg Chem. 2026 Jun 18;65(26):15164–73. doi: 10.1021/acs.inorgchem.6c02082 (PMC13343509; doi:10.1021/acs.inorgchem.6c02082)
Supplement: Supplementary file 3 [file ic6c02082_si_003.pdf]

# The effect of N-donor ligands on activation of $[Ru(tpy)(L{-}py)(AcCN)]^{2+}$ pre-catalysts in $CO_2$ reduction reaction electrocatalysis

Dr. Maurício P. Franco, Giovana V. Teixeira, and Prof. Dr. André L. B. Formiga*

Instituto de Química – UNICAMP, Caixa Postal: 6154, CEP: 13083-970, Campinas-SP

E-mail: formiga@unicamp.br

Phone: +55 19 3521 3420

**Table S1:** Energy gap between triplet and singlet states ($\Delta_{T-S}$) in eV for *cis* and *trans* isomers of $[Ru(tpy)(py\text{-}L)AcCN]^0$ and $[Ru(tpy)(py\text{-}L)]^0$ calculated with TPSSh/def2-svp with D3BJ dispersion corrections and acetonitrile as implicit solvent.

| | $\Delta_{T-S}$ (eV) – $[Ru(tpy)(py\text{-}L)$***AcCN***$]^0$ | | $\Delta_{T-S}$ (eV) – $[Ru(tpy)(py\text{-}L)]^0$ | |
|---|---|---|---|---|
| **Ligand** | **cis** | **trans** | **cis** | **trans** |
| **bpy** | -0.27 | | 0.28 | |
| **NHC** | -0.17 | -0.23 | 0.61 | 0.32 |
| **123-trz** | -0.16 | -0.24 | 0.40 | 0.46 |
| **pyrr** | -0.24 | -0.27 | 0.42 | 0.40 |
| **oz** | -0.30 | -0.27 | 0.27 | 0.31 |
| **prz** | -0.19 | 0.13 | 0.47 | 0.53 |

a)

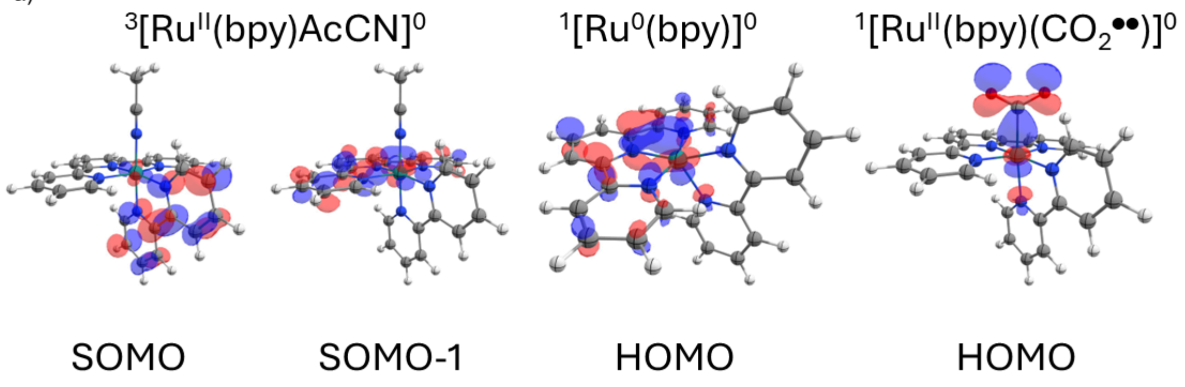

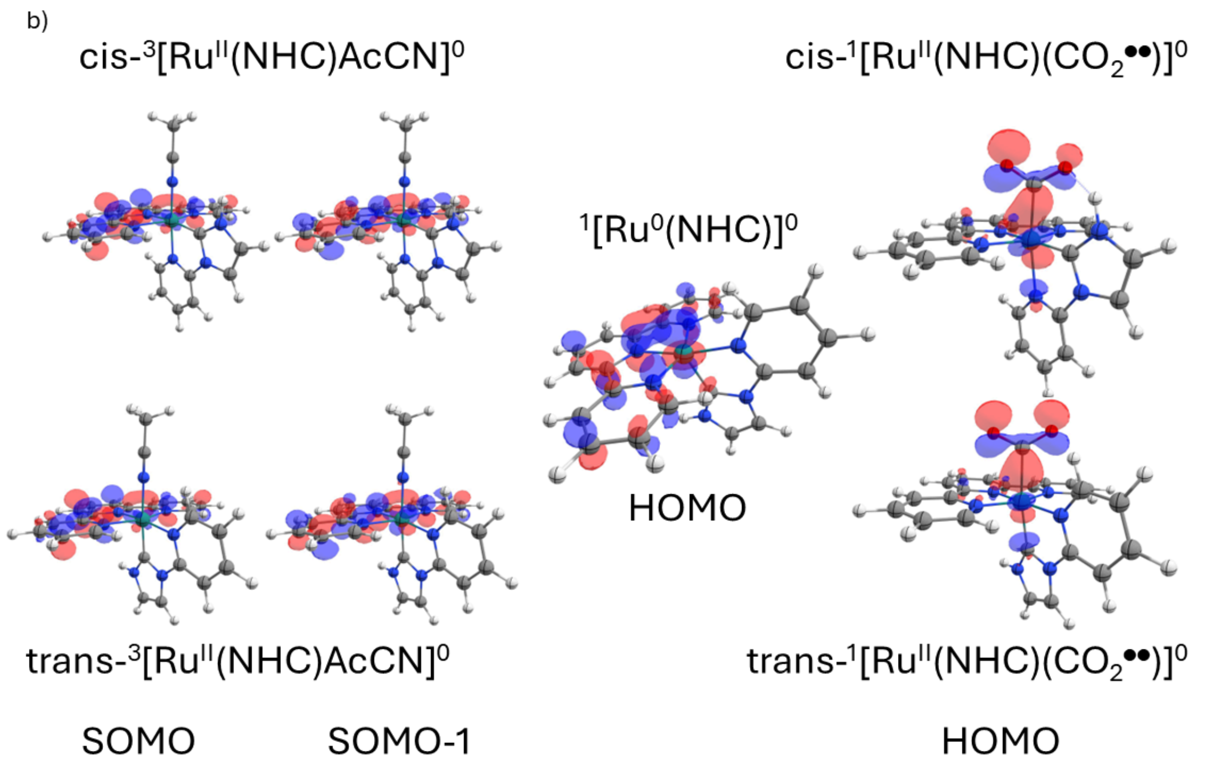
b)
cis-$^{3}$[Ru$^{II}$(NHC)AcCN]$^{0}$
cis-$^{1}$[Ru$^{II}$(NHC)($CO_2$••)]$^{0}$
$^{1}$[Ru$^{0}$(NHC)]$^{0}$
HOMO
trans-$^{3}$[Ru$^{II}$(NHC)AcCN]$^{0}$
trans-$^{1}$[Ru$^{II}$(NHC)($CO_2$••)]$^{0}$
SOMO
SOMO-1
HOMO

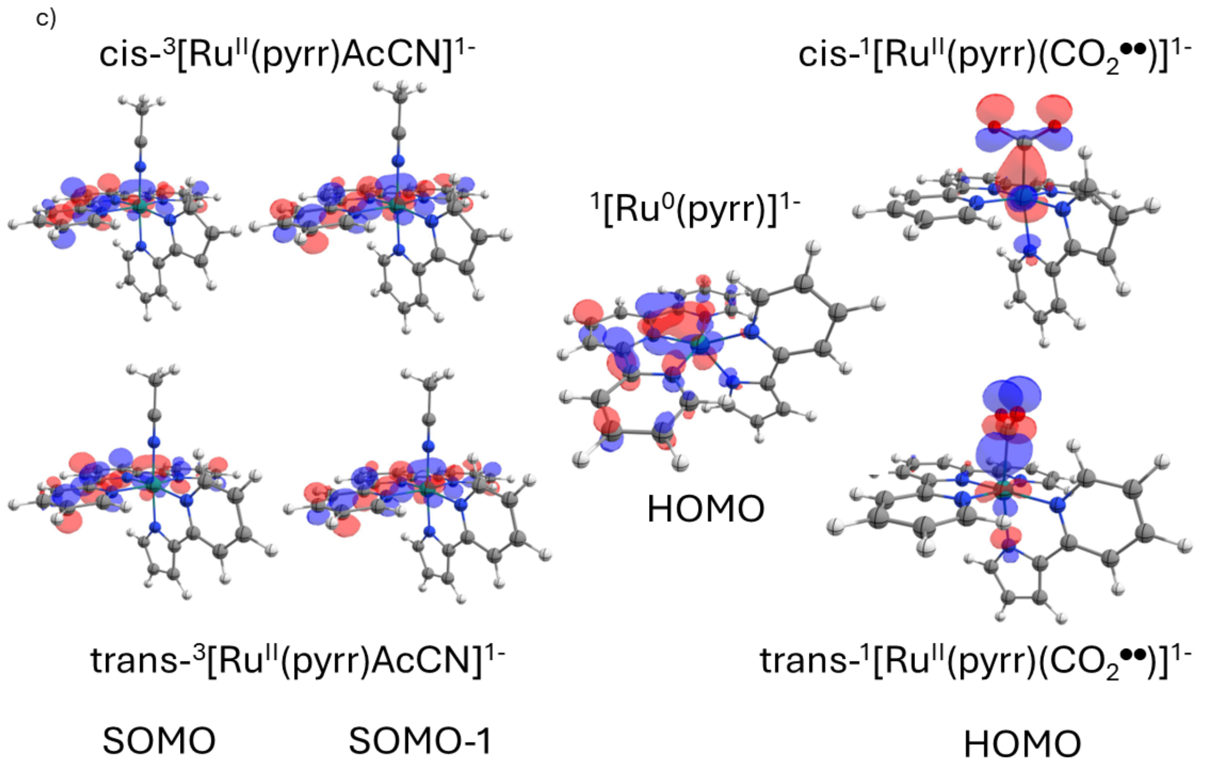
c)
cis-$^{3}$[Ru$^{II}$(pyrr)AcCN]$^{1-}$
cis-$^{1}$[Ru$^{II}$(pyrr)($CO_2$••)]$^{1-}$
$^{1}$[Ru$^{0}$(pyrr)]$^{1-}$
HOMO
trans-$^{3}$[Ru$^{II}$(pyrr)AcCN]$^{1-}$
trans-$^{1}$[Ru$^{II}$(pyrr)($CO_2$••)]$^{1-}$
SOMO
SOMO-1
HOMO

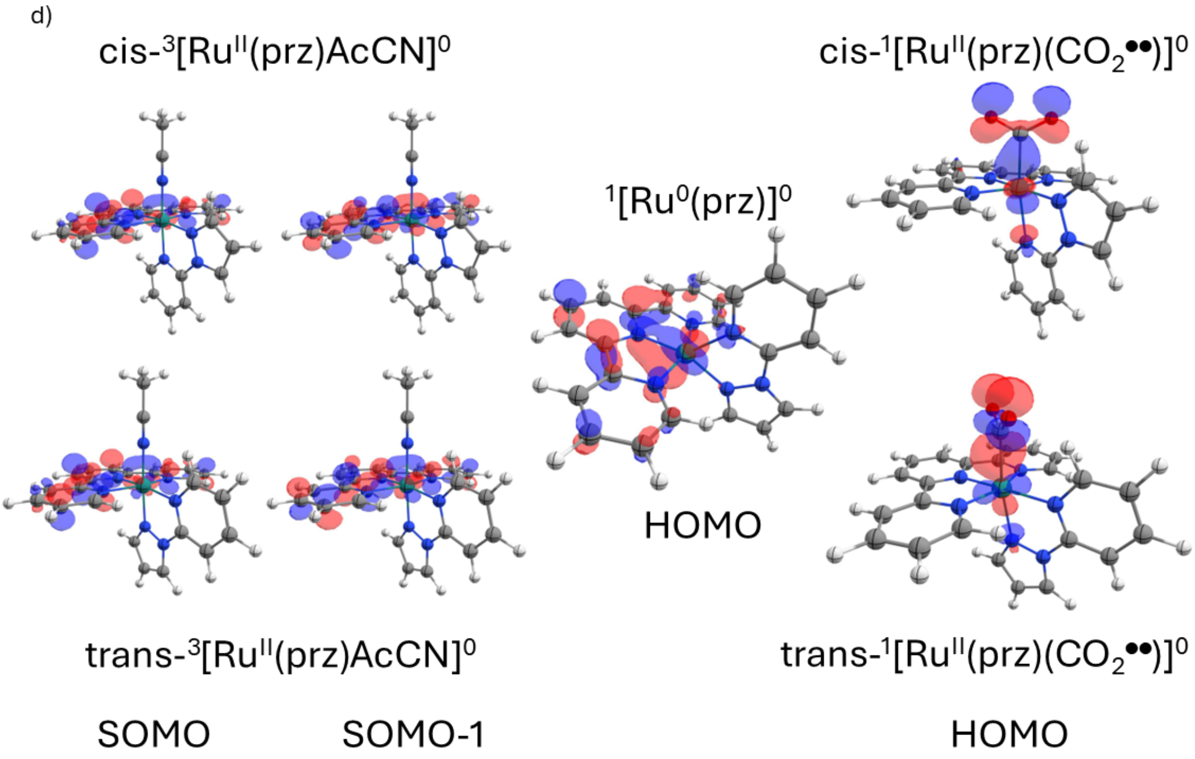
d)
cis-$^{3}$[Ru$^{II}$(prz)AcCN]$^{0}$
cis-$^{1}$[Ru$^{II}$(prz)($CO_2^{\bullet\bullet}$)]$^{0}$
$^{1}$[Ru$^{0}$(prz)]$^{0}$
HOMO
trans-$^{3}$[Ru$^{II}$(prz)AcCN]$^{0}$
trans-$^{1}$[Ru$^{II}$(prz)($CO_2^{\bullet\bullet}$)]$^{0}$
SOMO
SOMO-1
HOMO

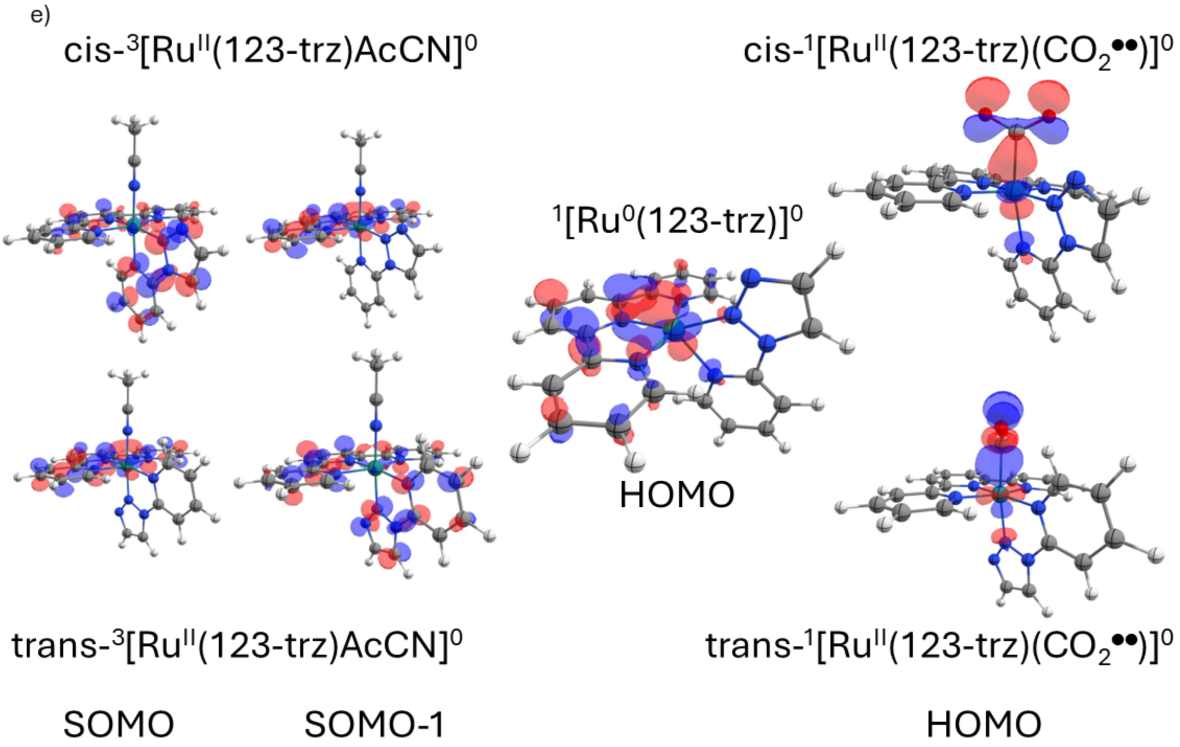
e)
cis-$^{3}$[Ru$^{II}$(123-trz)AcCN]$^{0}$
cis-$^{1}$[Ru$^{II}$(123-trz)($CO_2^{\bullet\bullet}$)]$^{0}$
$^{1}$[Ru$^{0}$(123-trz)]$^{0}$
HOMO
trans-$^{3}$[Ru$^{II}$(123-trz)AcCN]$^{0}$
trans-$^{1}$[Ru$^{II}$(123-trz)($CO_2^{\bullet\bullet}$)]$^{0}$
SOMO
SOMO-1
HOMO

f)

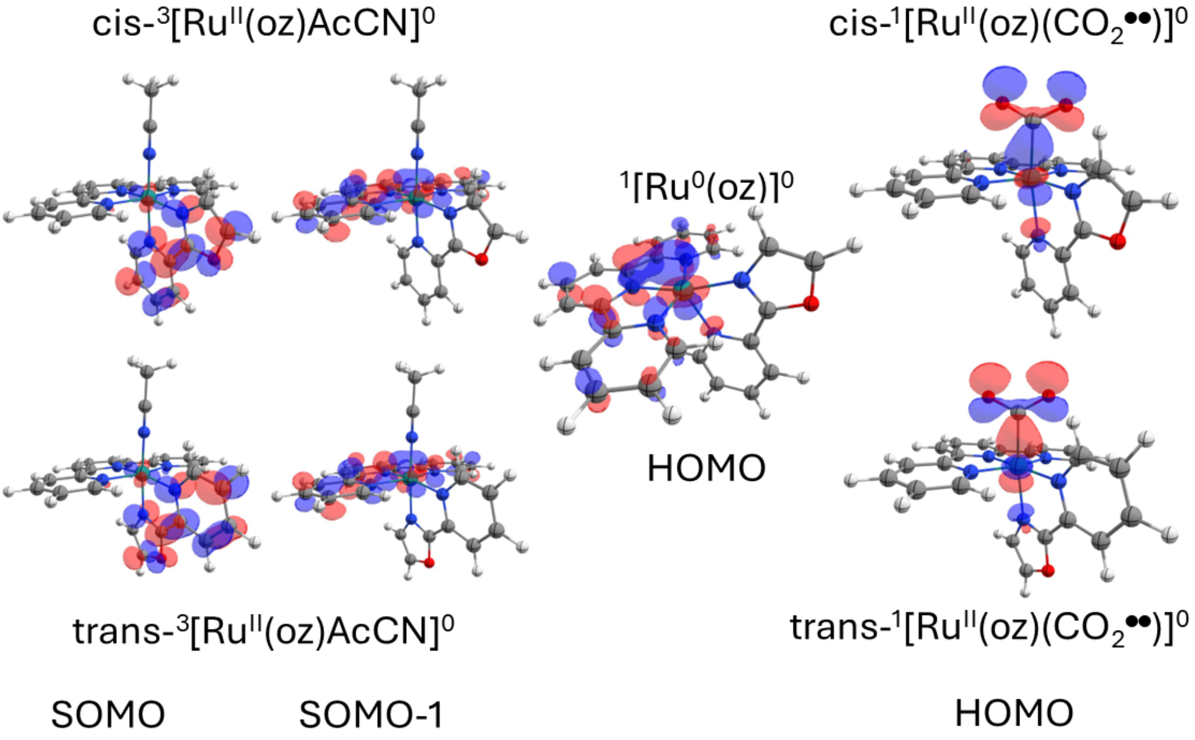


**Figure S1a-f.** Surface plots of the orbital(s) occupied by the two electrons after reductions and the orbitals involved in the electron transfer from cis/trans-$^3$[Ru(tpy)(py-L)(AcCN)]$^0$ to $^1$[Ru(tpy)(bpy)]$^0$ followed by cis/trans-$^1$[Ru(tpy)(py-L)($CO_2$)]$^0$ complex. a) **bpy**, b) **NHC**, c) **pyrr**, d) **prz**, e) **123-trz** and f) **oz** showing the electron density with isovalue = 0.05 electrons/Å³.

**Table S2:** Reduction Potential in Volt (V) calculated with TPSSh def2-tzvp//def2-svp with D3BJ dispersion corrections and acetonitrile as implicit solvent for (cis/trans)-$^2$[Ru(tpy)(py-L)]$^+$ + $e^-$ → trans-$^1$[Ru(tpy)(py-L)]$^0$.

| Ligands | Me-NHC | | NHC | | pyrr | | prz | |
|---|---|---|---|---|---|---|---|---|
| Isomers | cis | trans | cis | trans | cis | trans | cis | trans |
| $^1E_2$ ($ML_5$) | -1.00 | -1.31 | -1.03 | -1.32 | -1.43 | -1.59 | -1.14 | -1.11 |

* Experimental reduction potential value for cis-[Ru(tpy)(py-MeBim)S]$^{2+}$ $E_1$ = -1.05 V *vs* NHE and $E_2$ = -1.31 V *vs* NHE and for trans-[Ru(tpy)(py-MeBim)S]$^{2+}$ $E_1$ = -1.06 V *vs* NHE and $E_2$ = -1.31 V *vs* NHE

For comparison with experimental data, calculations were done for the methyl ligand Me-NHC as well. Results were compared against the benzoimidazolium ligand reported in

the literature.[1] The structures of both ligands are presented in Figure S1. The Me-NHC behaves similarly to the NHC with the first and second reductions localized at the tridentate ligand and the most stable spin state for both doubly reduced compounds is the triplet state. Comparison data on reduction potentials are presented in Table S1.

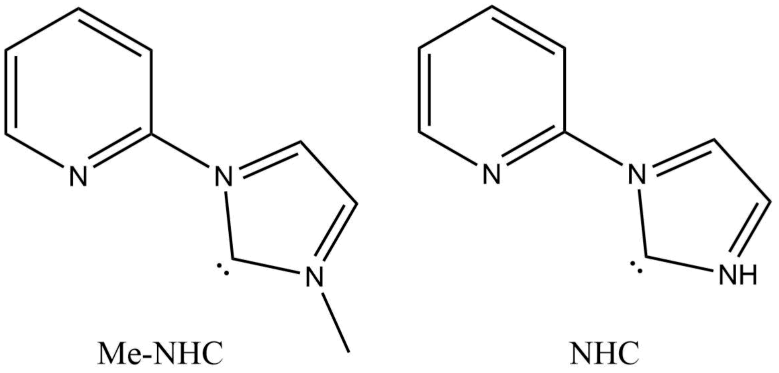


**Figure S2.** Structures of ligands Me-NHC and NHC

**Table S3:** Reduction Potential in Volt (V) for *cis* and *trans* isomers of $[Ru(tpy)(py\text{-}L)S]^{2+}$ calculated with TPSSh/def2-svp with D3BJ dispersion corrections and acetonitrile as implicit solvent.

| Ligands | Me-NHC | | NHC | |
|---|---|---|---|---|
| Isomers | cis | trans | cis | trans |
| $^{2}E_1$ | -1.07 | -1.11 | -1.07 | -1.11 |
| $^{3}E_2$ | -1.76 | -1.78 | -1.76 | -1.79 |
| $^{1}E_2$ | -1.95 | -2.03 | -1.93 | -2.02 |

* Experimental reduction potential value for cis-$[Ru(tpy)(py\text{-}MeBim)S]^{2+}$ $E_1$ = -1.05 V *vs* NHE and $E_2$ = -1.31 V *vs* NHE and for trans-$[Ru(tpy)(py\text{-}MeBim)S]^{2+}$ $E_1$ = -1.06 V *vs* NHE and $E_2$ = -1.31V *vs* NHE

Reduction potentials were compared to the experimental results presented by Gonell et. al.[1] and values for the first reductions are in good agreement with the experimental values for the benzomidazolium ligand. However, the second theoretical reduction is 400 mV

lower than the experimental observation, suggesting that the species reduced at the cyclic voltammetry is not the $[Ru(tpy)(py\text{-}NHC)S]^+$.

Considering the dissociation reaction at the first reduction as $[Ru(tpy)(py\text{-}L)S]^+ \rightarrow [Ru(tpy)(py\text{-}L)]^+ + S$ and the theoretical second reduction potential for the pentacoordinate species $[Ru(tpy)(py\text{-}L)]^+$ is $E_2$ = -1.32 V *vs* NHE for NHC and $E_2$ = -1.31 V for the Me-NHC, which is a better fit for the experimental results reported by Gonell *et. al.*.[1] Therefore, dissociation energies ($\Delta G_D$) were calculated from one-electron reduction species($\Delta G_{D1}$) and the values calculated for $\Delta G_{D1}$ were 8.5 and 14.6 kcal/mol for the trans and cis isomers respectively (Table S2), indicating that the trans isomer has a lower dissociation energy. Attempts to find the transition state for the one-electron reduction $[Ru(tpy)(py\text{-}L)S]^+$ were unsuccessful and the activation energy for this step were not found.

**Table S4:** Dissociation energies (D) in $\Delta G$ for the $[Ru(tpy)(py\text{-}L)S]^+$ in kcal/mol for both isomers and distortion parameter $\tau_5$ for the pentacoordinate complex $[Ru(tpy)(py\text{-}L)]^0$.

| Ligands | Me-NHC | | NHC | |
|---|---|---|---|---|
| Isomers | cis | trans | cis | trans |
| $\Delta G_{D1}$ | 14.6 | 8.5 | 15.5 | 9.6 |
| $\Delta G_{D2}$ | -3.0 | -2.4 | -1.4 | -1.3 |
| $\Delta G^{\ddagger}_{D2}$ | 14.0 | 7.8 | 14.8 | 7.9 |

The doubly reduced pentacoordinated complex of the Me-NHC ligand favors a closed-shell singlet over a triplet state given the $\Delta_{T\text{-}S}$ of 0.64 eV and 0.34 eV, positive value indicates a singlet state, for the cis and trans isomers, respectively.

After acetonitrile dissociation, both cis and trans isomers produce the same pentacoordinate complex, with geometries between square pyramidal (SP) and trigonal bipyramidal (TBP) identified by the $\tau_5$ distortion parameter, presented in table S2. This parameter is determined by the angles $\Phi$ (N-Ru-N) between tpy and either N on py or L on the bidentate ligand, whichever is higher, and admitting the angle between nitrogens in tpy is 180° since in either geometry they must be in an axial position in BPT or the equatorial plane in the SP geometry. Figure S2 presents the pentacoordinate complex for Me-NHC ligand and a comparison in $\Phi$ for Me-NHC and NHC ligands.

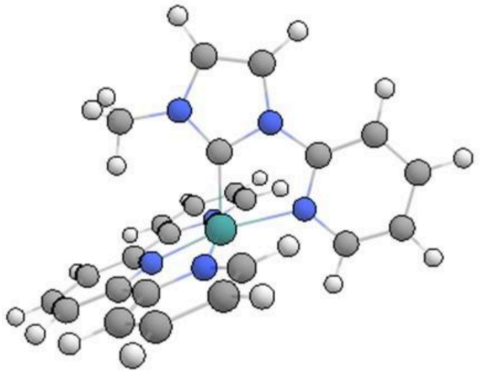

| | Me-NHC | NHC |
|---|---|---|
| $\phi^{py}$ | 167.5 | 167 |
| $\phi^{L}$ | 116.2 | 117 |

**Figure S3:** Pentacoordinate geometry for Me-NHC ligand and respective angles

Carbon dioxide association to the pentacoordinate complex was calculated presuming the formation of cis and trans isomers. Results are presented in Table S4 and they show a faster reaction than the dissociation, with smaller energy barriers. The transition state shows $CO_2$ association through the approximation of carbon towards ruthenium.

**Table S5:** Association reaction energies ($\Delta_R G_A$) and activation barrier ($\Delta G^{\ddagger}_A$) in kcal/mol for both isomers, the *cis* : *trans* proportion of metallocarboxylate isomers.

| Ligands | Me-NHC | | NHC | |
|---|---|---|---|---|
| Isomers | cis | trans | cis | trans |
| $\Delta_R G_A$ | -8.3 | -7.6 | -14.9 | -6.8 |
| $\Delta G^{\ddagger}_A$ | 9.8 | 4.8 | 6.8 | 4.2 |
| $\tau_5$ | 0.21 | | 0.22 | |
| cis : trans | 1 : 4624.1 | | 1 : 57.4 | |

This step can lead to cis and trans isomerization and by using the Eyring equation the cis : trans ratio was calculated and is presented in Table S4. The pentacoordinate complex's geometry directly impacts this ratio, and the closer the pentacoordinate geometry is to square pyramidal, the higher the isomer excess.

For the Me-NHC the trans isomer has a considerably lower $\Delta G^{\ddagger}_A$ than the Me-NHC cis isomer, on the other hand, the cis isomer presents the highest association barrier comparing the methyl system and the NHC model. The combination of the electronic effect of the methyl bonded to the nitrogen and steric hindrance of the methyl group located close

to the vacant site where $CO_2$ association causes a slight change of $\Delta G^{\ddagger}_{A}$ both configurations and a significant change in the $\Delta_R G_A$ for the cis configuration, however the ratio tendency compared to the model is maintained.

Microkinetics modelling (MKM) was done using COPASI 4.20 software. The chemical equations used to model it were

$$\text{cis/trans-}[Ru(tpy)(py{-}L)(AcCN)]^0 = [Ru(tpy)(py{-}L)]^0 + CH_3CN \quad (1)$$

$$[Ru(tpy)(py{-}L)]^0 + CO_2 = \text{cis-}[Ru(tpy)(py{-}L)(CO_2^{\bullet\bullet})]^0 \quad (2)$$

$$[Ru(tpy)(py{-}L)]^0 + CO_2 = \text{trans-}[Ru(tpy)(py{-}L)(CO_2^{\bullet\bullet})]^0 \quad (3)$$

Microkinetic modeling for the py-L complexes closely resembles that of the bpy system, even in the occurrence of competing cis and trans $CO_2$ addition pathways (Fig. S5), because the kinetic and thermodynamic products are identical. In contrast, for the NHC system the kinetic and thermodynamic products differ: the trans isomer is formed first due to a lower activation barrier, whereas the cis isomer is the most thermodynamically stable species(Table 3).

a)

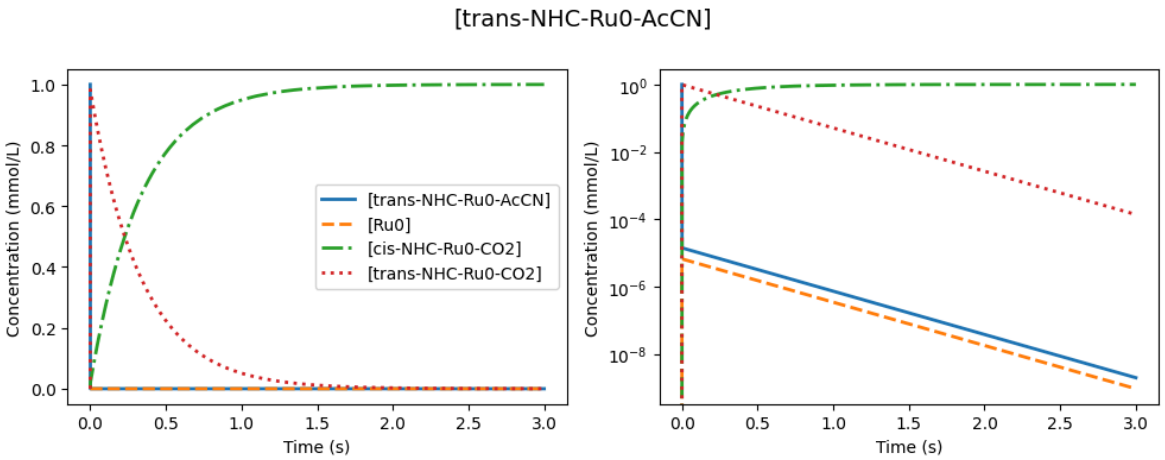


b)

[cis-pyrr-Ru0-AcCN]

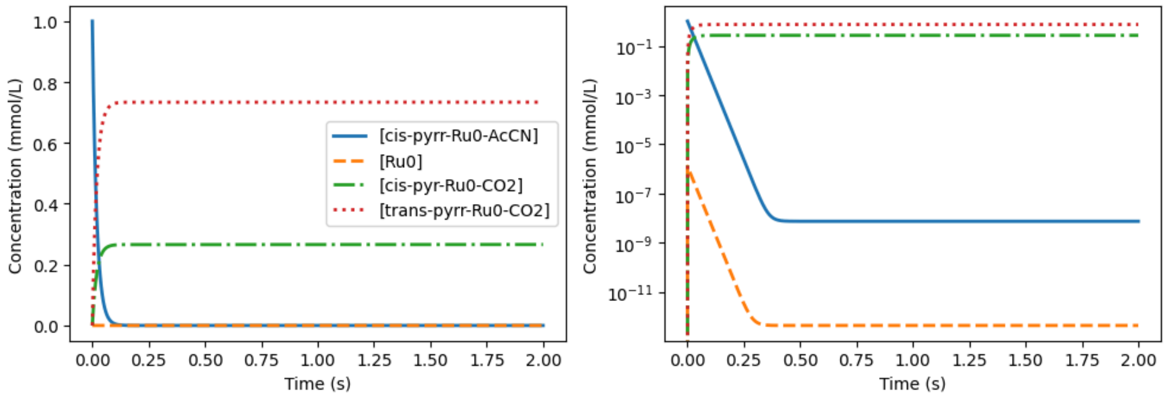


c)

[trans-pyrrr-Ru0-AcCN]

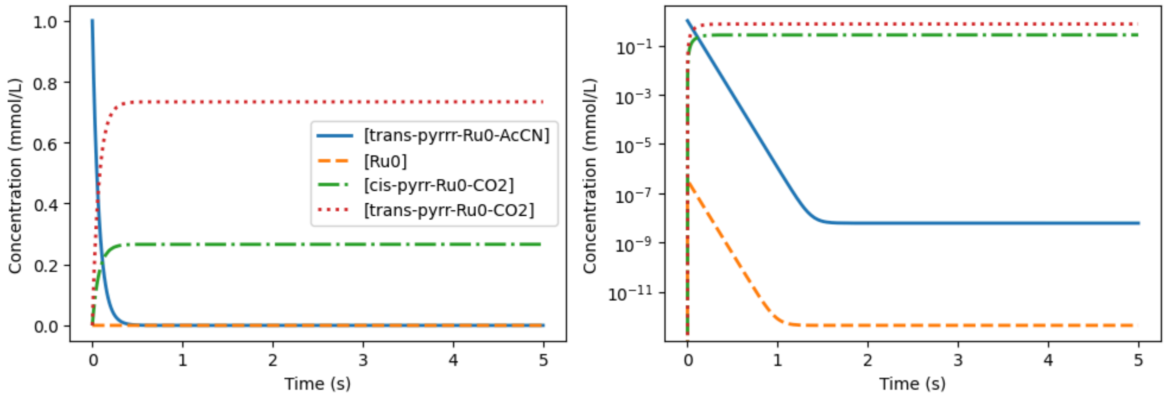


d)

[cis-prz-Ru0-AcCN]

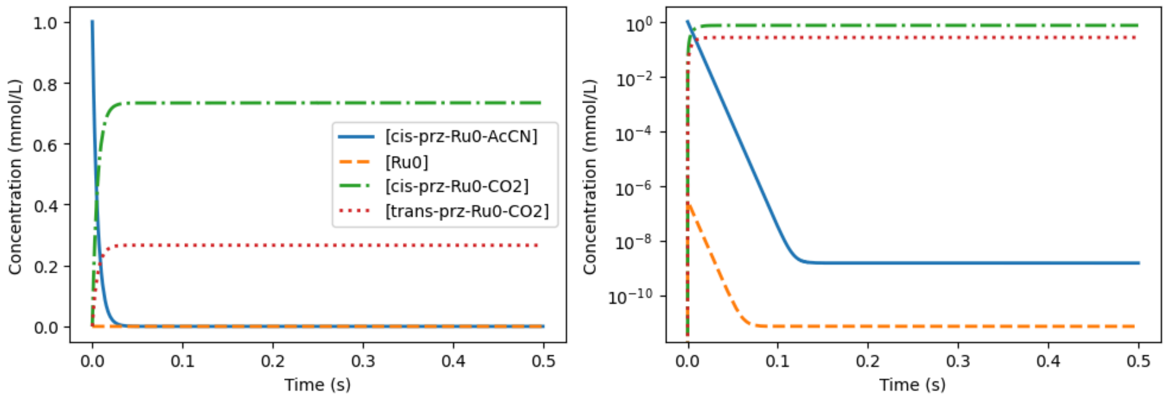


e)

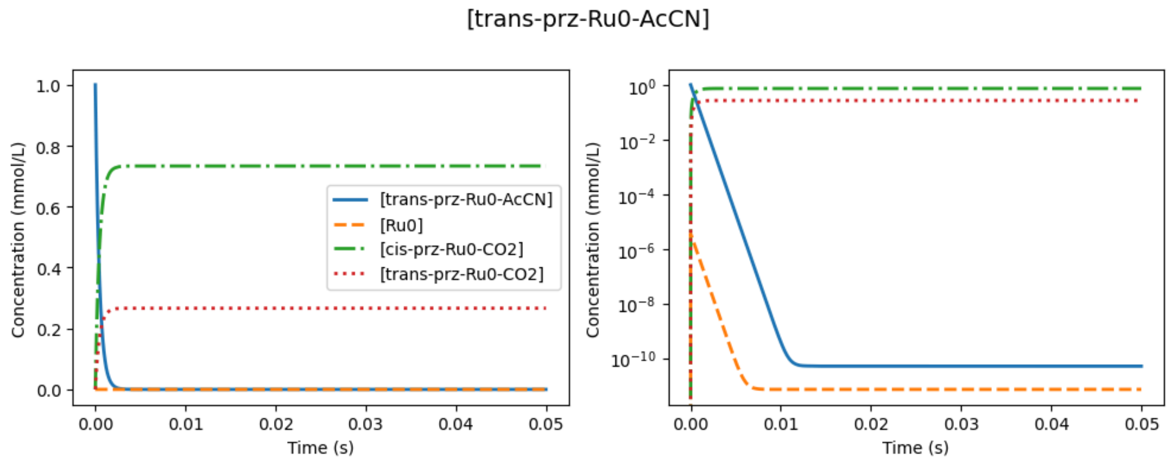
[trans-prz-Ru0-AcCN]
[trans-prz-Ru0-AcCN]
[Ru0]
[cis-prz-Ru0-CO2]
[trans-prz-Ru0-CO2]
Concentration (mmol/L)
Time (s)

f)

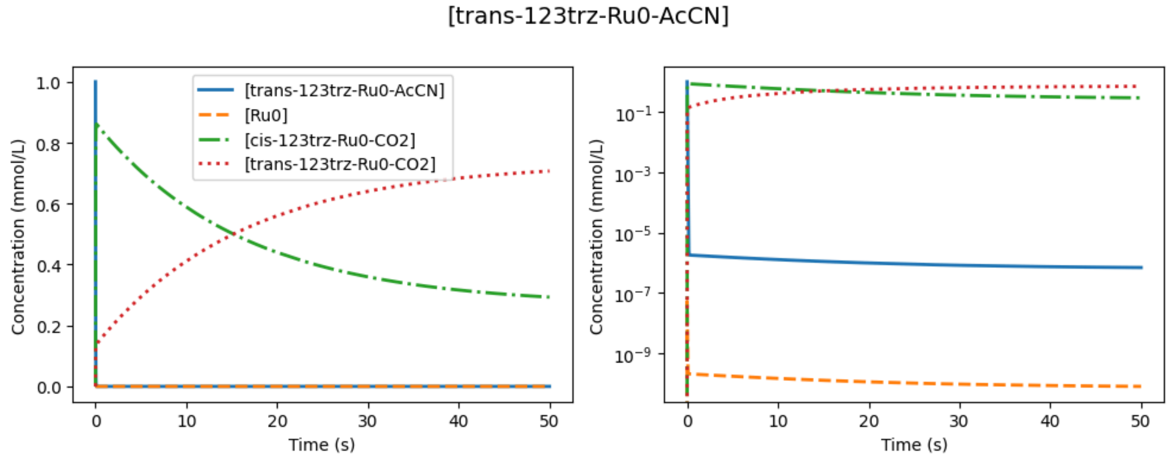
[trans-123trz-Ru0-AcCN]
[trans-123trz-Ru0-AcCN]
[Ru0]
[cis-123trz-Ru0-CO2]
[trans-123trz-Ru0-CO2]
Concentration (mmol/L)
Time (s)

g)

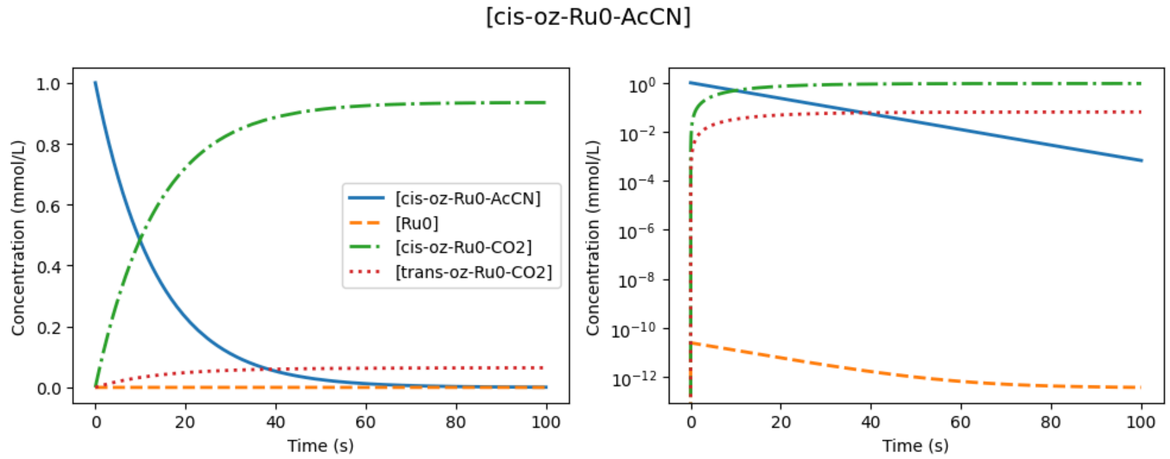
[cis-oz-Ru0-AcCN]
[cis-oz-Ru0-AcCN]
[Ru0]
[cis-oz-Ru0-CO2]
[trans-oz-Ru0-CO2]
Concentration (mmol/L)
Time (s)

h)

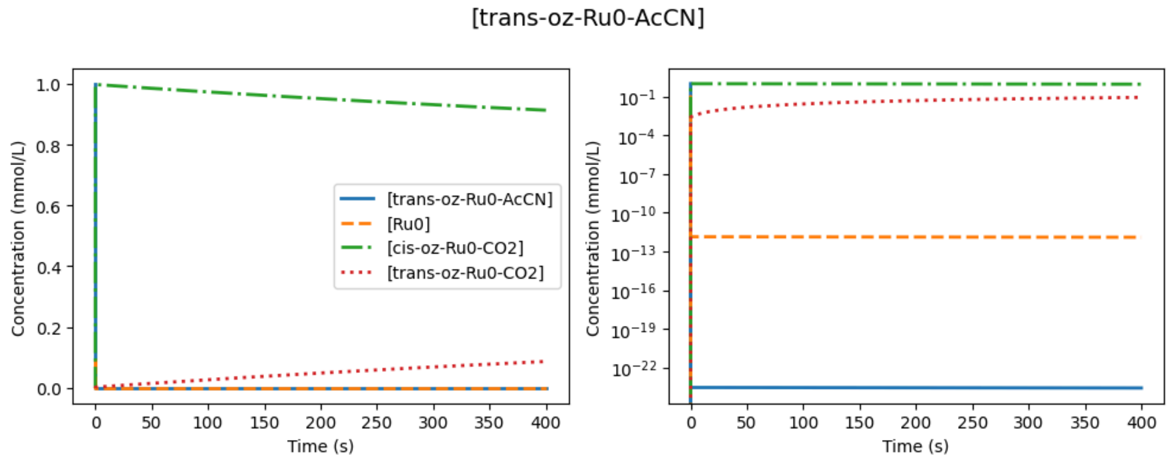


Figure S4: Microkinetic simulation of the cis/trans-[Ru(tpy)(py–L)(AcCN)]00/CO22 system obtained with COPASI. Left: concentration profiles on a linear scale; right: same data on a logarithmic y-axis. Initial conditions: [cis/trans-Ru(tpy)(py–L)(AcCN)]$^0$ = 1.0 mmol/L, [$CO_2$]= 0.28 mol/L; [AcCN] = 19.15 mol/L rate constants derived from DFT free energies via Eyring's equation.

Also, since the dissociation is more favorable than the bpy counterpart, the concentration of the Ru(0) species rises up to $10^{-4}$ mol/L, but only for simulations of $10^{-6}$ second. When considering only AcCN dissociation and reversible $CO_2$ addition, the trans isomer is preferentially produced at short times, while longer reaction times show accumulation of the thermodynamic (cis) product as a result of rapid equilibrium between the Ru(0) species and the trans-Ru–$CO_2$ species(Fig. S5a-b). However, experimental results indicate that the cis-Ru–$CO_2$ isomer is not observed at any stage during controlled electrolysis,[1] implying rapid consumption of the trans-Ru–$CO_2$ intermediate, which prevents its isomerization to the thermodynamic product.

**References**


[1] Gonell, S.; Massey, M. D.; Moseley, I. P.; Schauer, C. K.; Muckerman, J. T.; Miller, A. J. The Trans Effect in Electrocatalytic CO2 Reduction: Mechanistic Studies of Asymmetric Ruthenium Pyridyl-Carbene Catalysts. Journal of the American Chemical Society 2019, 141, 6658–6671.

[2] Pavlishchuk, V. V.; Addison, A. W. Inorganica Chimica Acta 2000, 298, 97–102.

Pavlishchuk V. V. and Addison, A. W. Conversion constants for redox potentials measured versus different reference electrodes in acetonitrile solutions at 25°C, Inorganica Chimica Acta, 2000, 298, 1, 97-102.
